# Supplementary material for: NFAT transcription factors are essential and redundant actors for leukemia initiating potential in T-cell acute lymphoblastic leukemia
Source: PLoS One. 2021 Jul 7;16(7):e0254184. doi: 10.1371/journal.pone.0254184 (PMC8263285; doi:10.1371/journal.pone.0254184)
Supplement: S1 Fig — (A) TEL-JAK2 transgenic mice were crossed and backcrossed with mice inactivated for Nfat1 to generate cohorts of TEL-JAK2+/0; Nfat1+/- (n = 17) and TEL-JAK2+/0; Nfat1-/- (n = 18) littermates. As reported previously, expression of the TEL-JAK2 fusion oncogene in mouse lymphoid lineage induced T-ALL with high penetrance. These cohorts were followed over time for T-ALL onset and mouse survival (log-rank test; ns: non-significant). (B) Mice carrying ICN1-induced T-ALL, a well characterized T-ALL model induced by activated NOTCH1, with the indicated genotypes (Nfat1+/+, n = 35; Nfat1-/-, n = 28) were followed over time for T-ALL onset and recipient mice survival (log-rank test; ns: non-significant). (PPTX) [file pone.0254184.s001.pptx]

## Slide 1
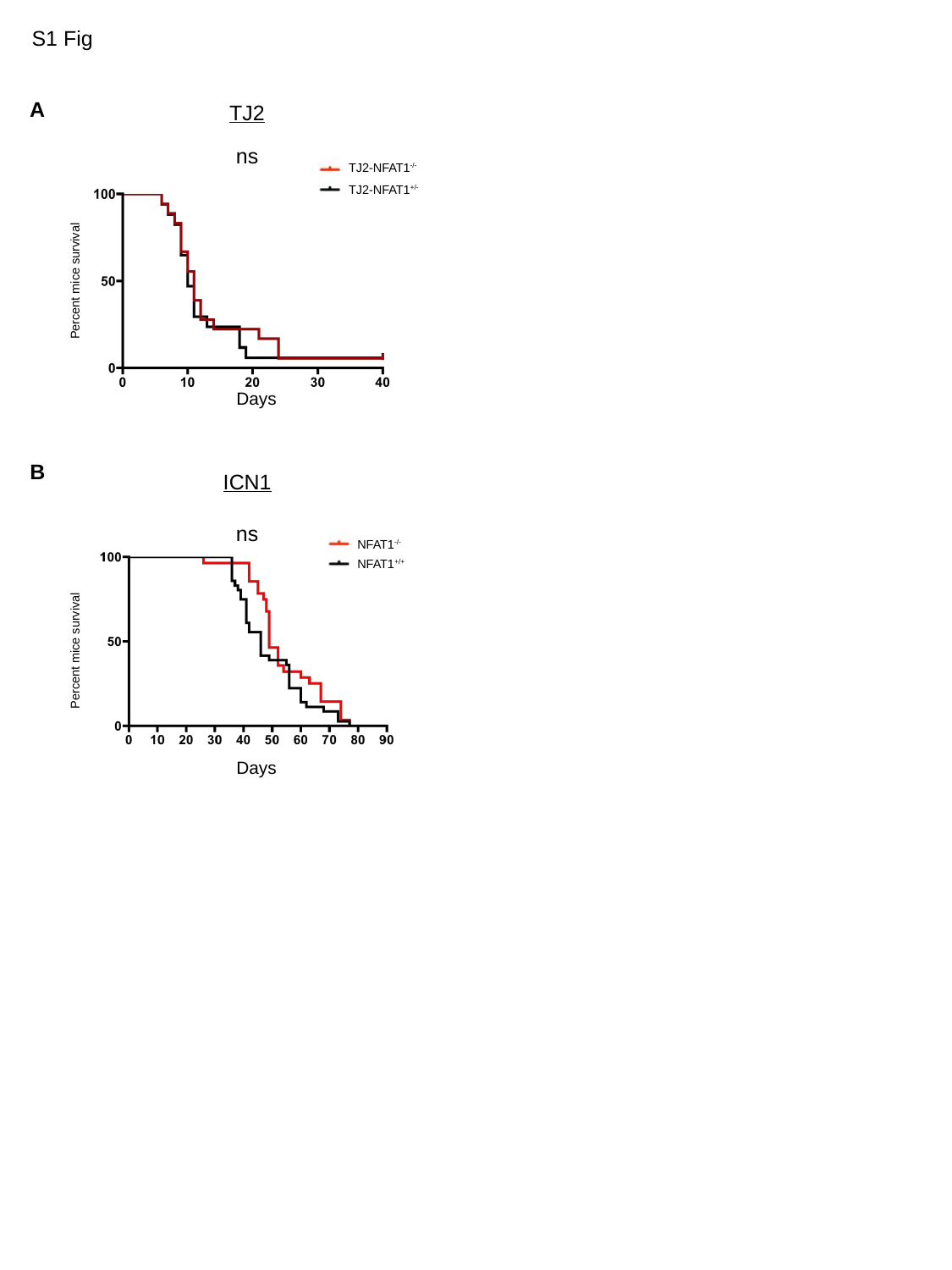

S1 Fig
A
TJ2
ns
TJ2-NFAT1-/-
TJ2-NFAT1+/-
Percent mice survival
Days
B
ICN1
ns
NFAT1-/-
NFAT1+/+
Percent mice survival
Days
