# Supplementary figures and images for: NFAT transcription factors are essential and redundant actors for leukemia initiating potential in T-cell acute lymphoblastic leukemia
Source: PLoS One. 2021 Jul 7;16(7):e0254184. doi: 10.1371/journal.pone.0254184 (PMC8263285; doi:10.1371/journal.pone.0254184)

## Slide 1
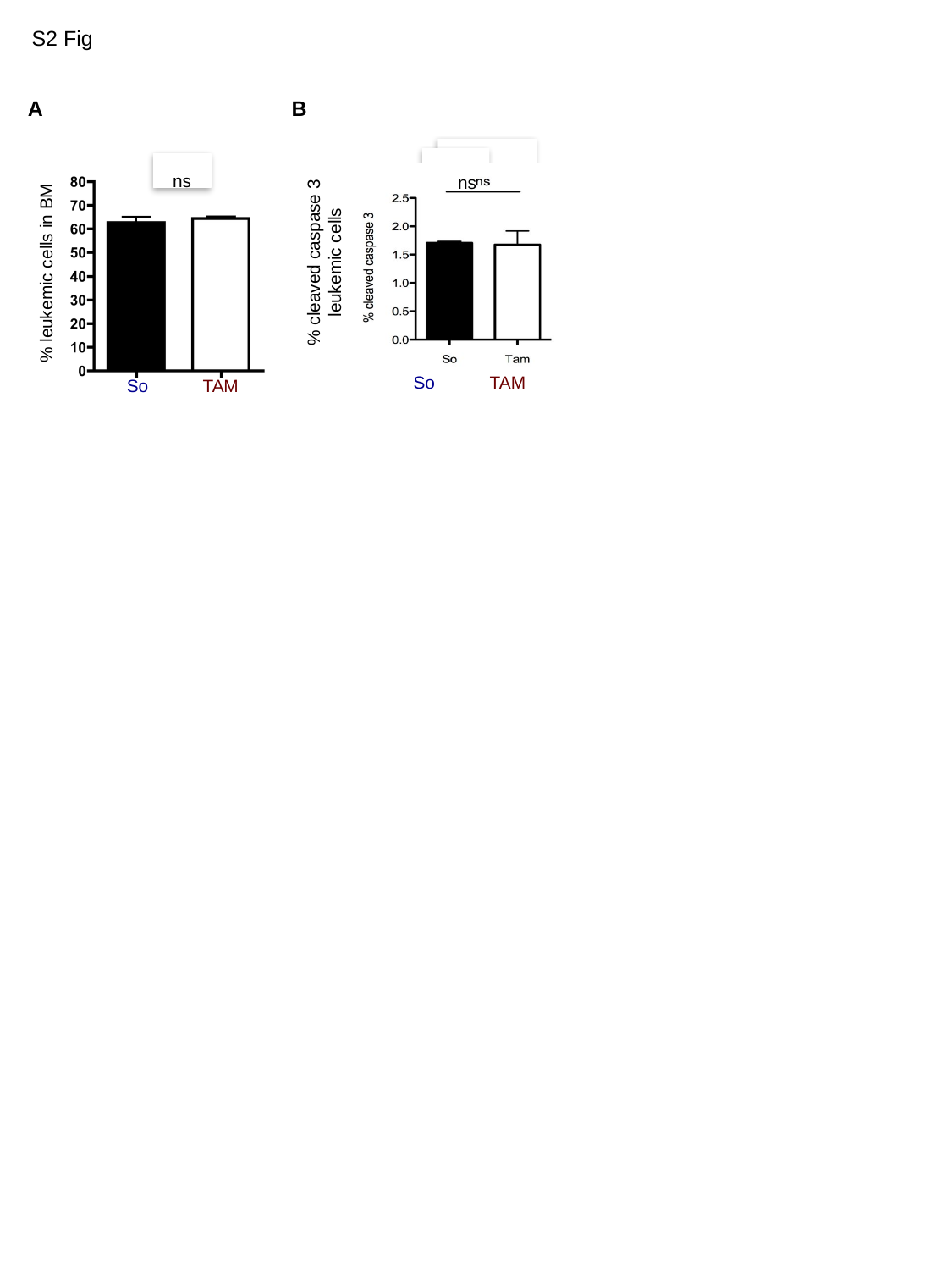

S2 Fig
A
B
ns
% leukemic cells in BM
ns
% cleaved caspase 3 leukemic cells
So
TAM
So
TAM

Supplement: S2 Fig — The Nfat-proficient and Nfat-deficient versions of T-ALL #21 were generated as described in Fig 1A. Mice were sacrificed when terminally ill, 2 days after the end of So and Tam treatments (1 and 2 in Fig 1A). (A) Leukemic burden (% tNGFR+ cells) in BM was analyzed by flow cytometry at the time mice were killed (data are represented as ± SEM; n = 3; Student’s t-test; ns: non-significant). (B) Apoptosis in leukemic cells described in (A) was analyzed by measuring caspase 3 activation by flow cytometry (data are represented as ± SEM; n = 3; Student’s t-test; ns: non-significant). (PPTX) [file pone.0254184.s002.pptx]

## Slide 1
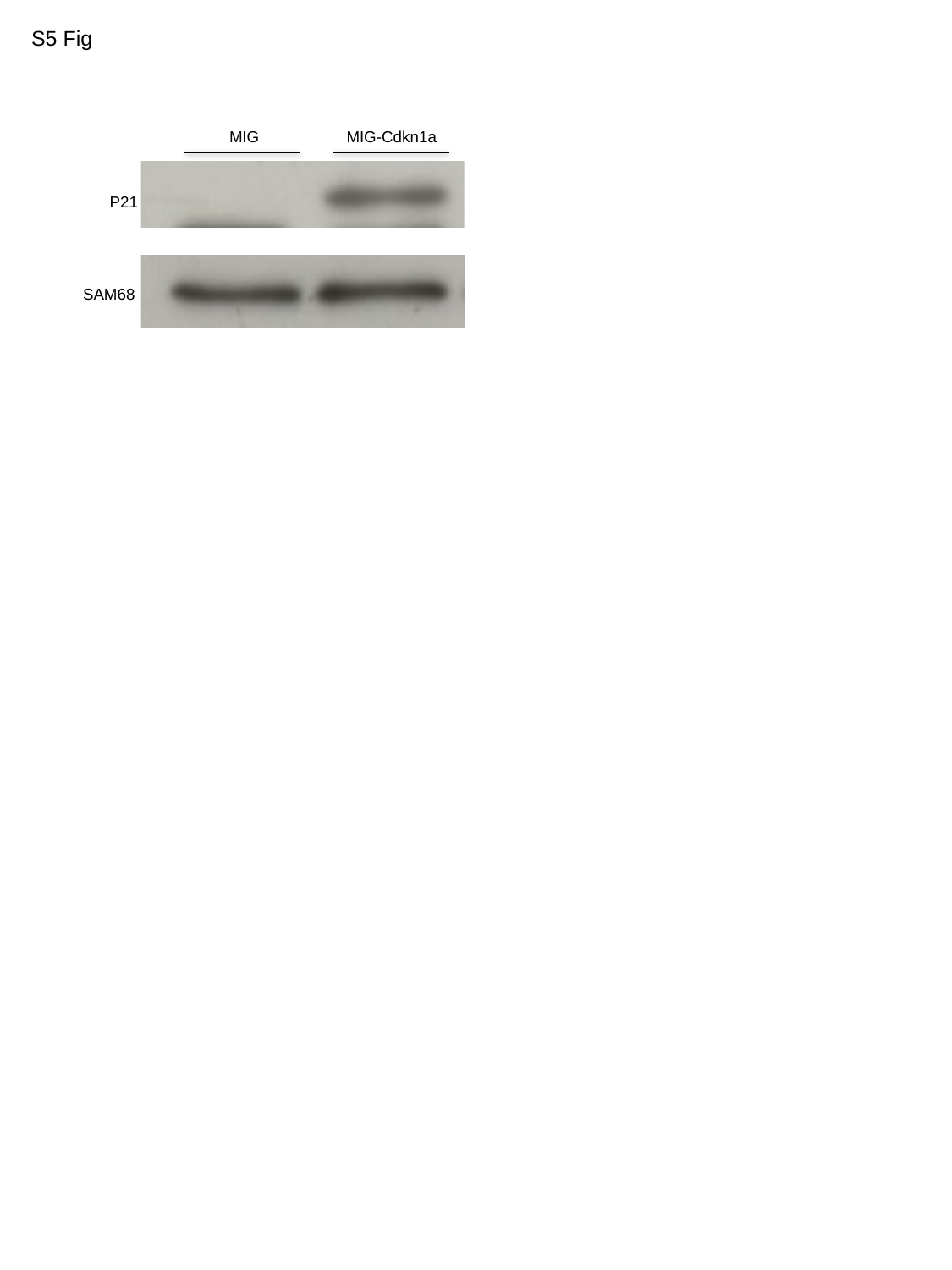

S5 Fig
MIG
MIG-Cdkn1a
P21
SAM68

Supplement: S5 Fig — Leukemic cells co-cultured on MS5 stromal cells for 2 days were analyzed by western blot for P21 expression. SAM68 is used as loading control. (PPTX) [file pone.0254184.s005.pptx]

## Slide 1
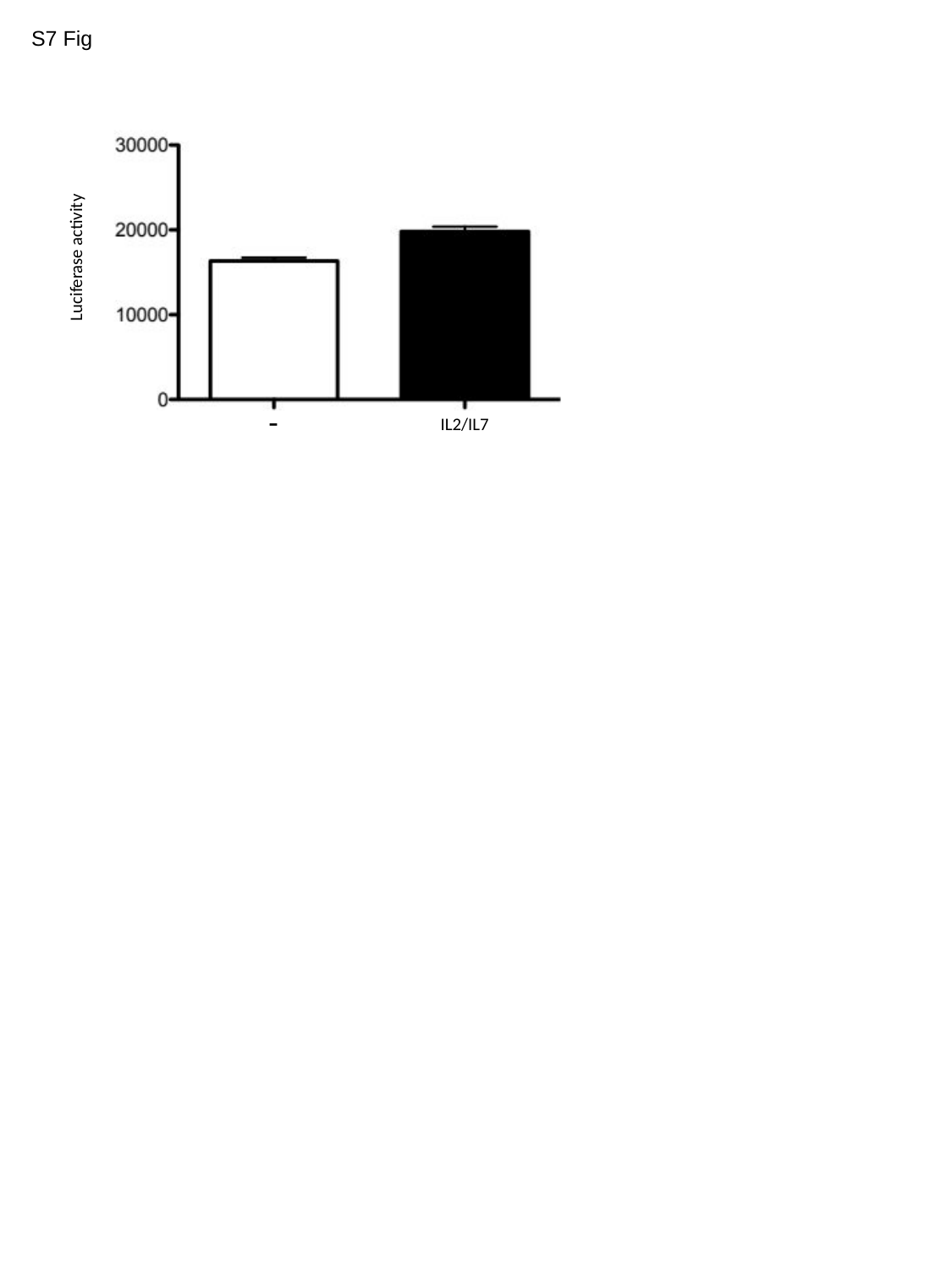

S7 Fig
Luciferase activity
-
IL2/IL7

Supplement: S7 Fig — Note the lack of induction of luciferase activity as compared to untreated cells. (PPTX) [file pone.0254184.s007.pptx]
