# Supplementary material for: NFAT transcription factors are essential and redundant actors for leukemia initiating potential in T-cell acute lymphoblastic leukemia
Source: PLoS One. 2021 Jul 7;16(7):e0254184. doi: 10.1371/journal.pone.0254184 (PMC8263285; doi:10.1371/journal.pone.0254184)
Supplement: S3 Fig — (A) Schematic representation of the experiment: Mice were injected with leukemic cells obtained from T-ALL ICN1; RCT2; NFAT1+/+; NFAT2+/+; NFAT4+/+. When BM leukemia burden reached about 10–15% leukemic cells in recipients, mice received 3 successive daily injection of either carrier solvent (So, n = 3) or tamoxifen (TAM, n = 6). Terminally ill mice from both groups were sacrificed 2 days later and leukemic cells from So-treated (blue label, 1) or Tam-treated (red label, 2) cells (106 cells/mouse) were transplanted in wild-type secondary recipients that were followed for leukemia recurrence. (B) Kaplan-Meier survival curve of recipient mice infused with 1x10^6^ T-ALL #RC2 cells. Mice were followed overtime for tumor recurrence and recipient mice survival. (C) Leukemic burden analysis of recipient mice (n = 3 for each group) infused with T-ALL #RC2 cells expressing NFAT factors Nfat-proficient (data are represented as ± SEM; n = 3; Student’s t test; ns: non-significant). (PPTX) [file pone.0254184.s003.pptx]

## Slide 1
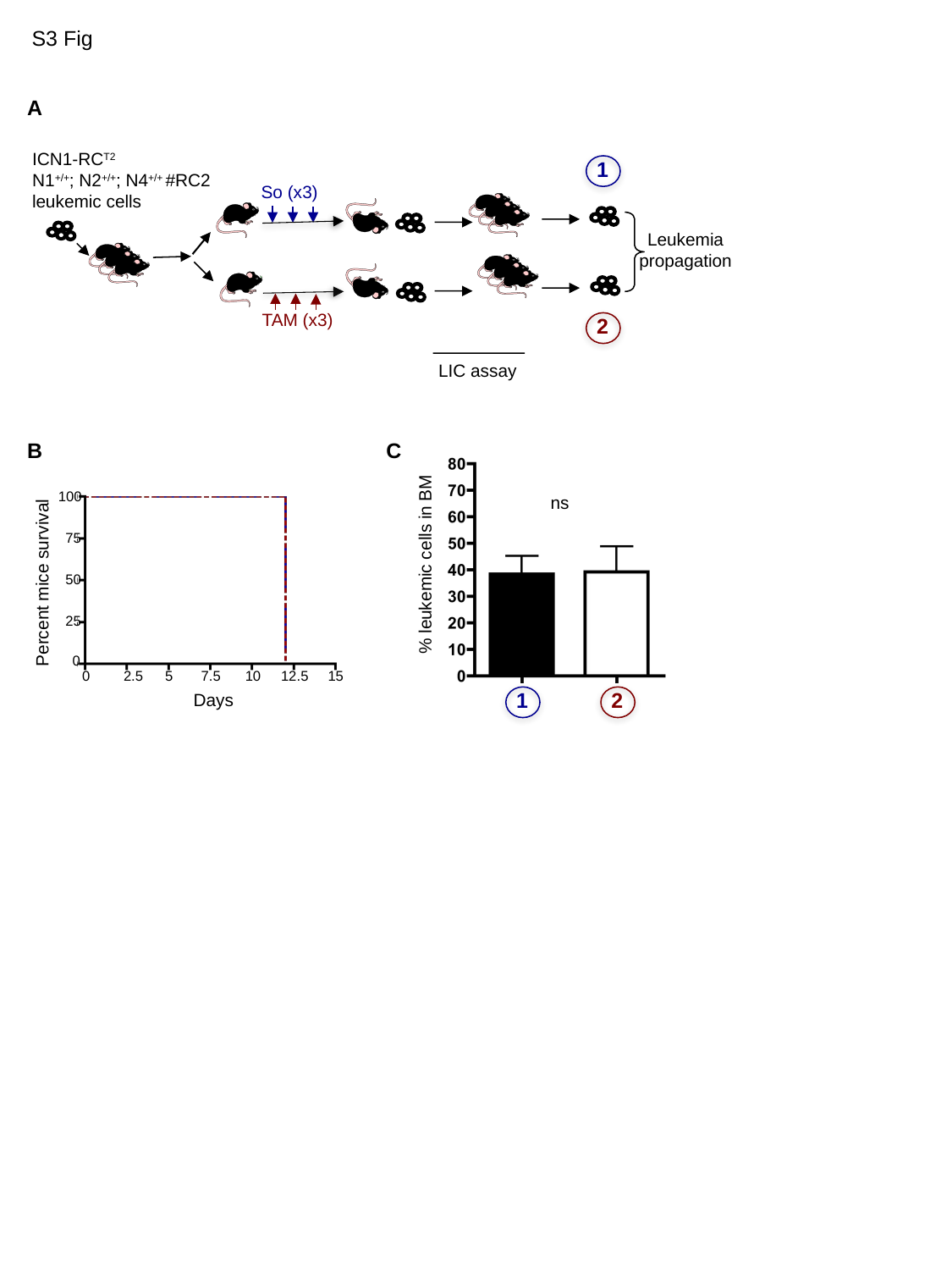

S3 Fig
A
ICN1-RCT2
N1+/+; N2+/+; N4+/+ #RC2
leukemic cells
1
So (x3)
Leukemia
propagation
TAM (x3)
2
LIC assay
B
C
Percent mice survival
Days
100
ns
75
% leukemic cells in BM
50
25
0
15
0
5
10
2.5
7.5
12.5
1
2
