# Supplementary material for: NFAT transcription factors are essential and redundant actors for leukemia initiating potential in T-cell acute lymphoblastic leukemia
Source: PLoS One. 2021 Jul 7;16(7):e0254184. doi: 10.1371/journal.pone.0254184 (PMC8263285; doi:10.1371/journal.pone.0254184)
Supplement: S4 Fig — (A) 5 days after ethanol or 4OHT treatment leukemic cells were analyzed for percentage of cells positive for cleaved caspase 3 (data are represented as mean ± SEM; n = 3; Student’s t-test; ns = not significant). (B) At the same time point EtOH and 4OHT-treated leukemic cells were re-seeded on fresh MS5 stromal cells and migration of individual cells (n = 30) recorded for 15 minutes by time-lapse video-microscopy. In the flower plot diagrams (left), the starting point of each track is placed at the axis origins. (C) In the right panel, velocity (μm/min) of leukemic cell was compared (data are represented as mean ± SEM; n = 3; Student’s t-test; ns = not significant). (PPTX) [file pone.0254184.s004.pptx]

## Slide 1
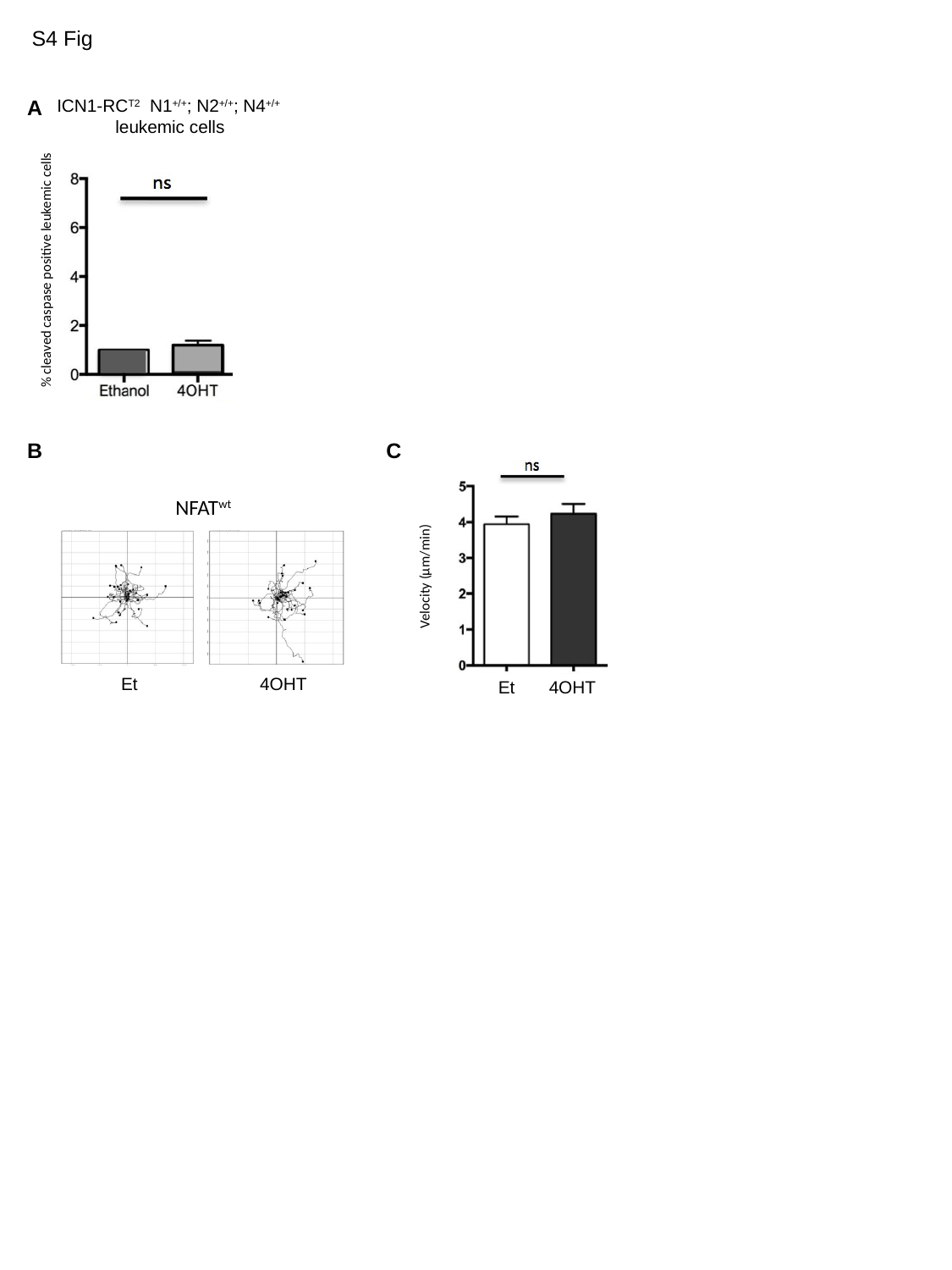

S4 Fig
A
ICN1-RCT2 N1+/+; N2+/+; N4+/+
leukemic cells
% cleaved caspase positive leukemic cells
B
C
NFATwt
Velocity (µm/min)
Et
4OHT
Et
4OHT
