# Supplementary material for: NFAT transcription factors are essential and redundant actors for leukemia initiating potential in T-cell acute lymphoblastic leukemia
Source: PLoS One. 2021 Jul 7;16(7):e0254184. doi: 10.1371/journal.pone.0254184 (PMC8263285; doi:10.1371/journal.pone.0254184)
Supplement: S6 Fig — MFI quantification obtained for independent mice of each group. Data are represented as mean ± SEM (n = 3 for control and n = 9 for ICN1; RCT2; Nfat1-/-; Nfat2fl/del; Nfat4fl/fl leukemic cells. ns, not significant; Student’s t test). (PPTX) [file pone.0254184.s006.pptx]

## Slide 1
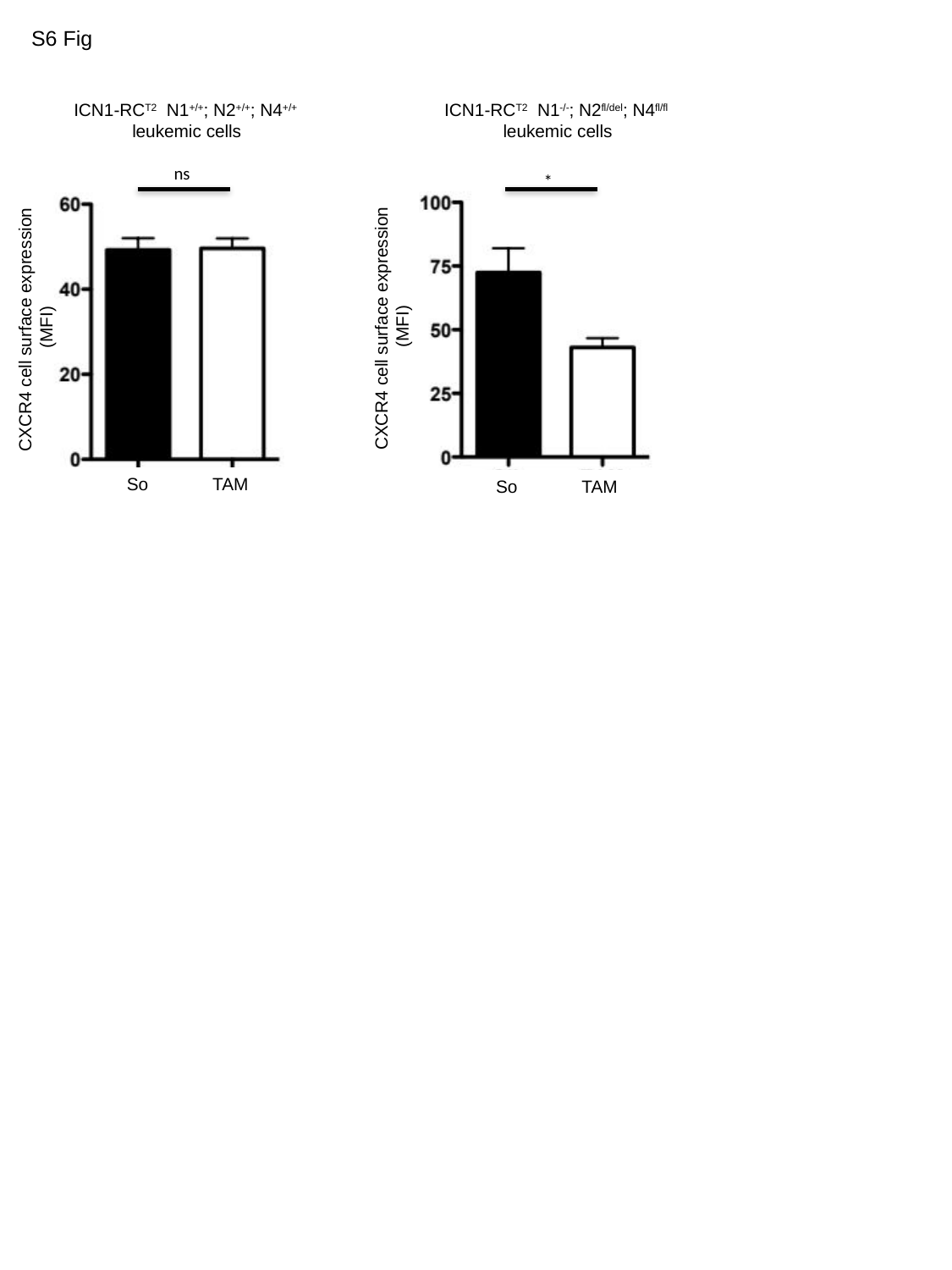

S6 Fig
ICN1-RCT2 N1-/-; N2fl/del; N4fl/fl
leukemic cells
ICN1-RCT2 N1+/+; N2+/+; N4+/+
leukemic cells
ns
*
CXCR4 cell surface expression
(MFI)
CXCR4 cell surface expression
(MFI)
So
TAM
So
TAM
