# Supplementary material for: NFAT transcription factors are essential and redundant actors for leukemia initiating potential in T-cell acute lymphoblastic leukemia
Source: PLoS One. 2021 Jul 7;16(7):e0254184. doi: 10.1371/journal.pone.0254184 (PMC8263285; doi:10.1371/journal.pone.0254184)
Supplement: S1 Table — (DOCX) [file pone.0254184.s008.docx]

**S1 Table.** Comparison of the leukemia initiating potential of ICN1; RC2; Nfat ^+/+^ T-ALL

| **Leukemia**  **id** | **Treatment of donor mice** | **Number of cells injected in recipients** | **Number of injected recipients** | **Number of leukemic recipients (time to death, days)** | | **Statistics** | **% leukemic cells in the BM ± SEM of recipients** | |
| --- | --- | --- | --- | --- | --- | --- | --- | --- |
| #RC2 | So | 4.10^6^ | 3 | | 3 (14 ;16 ;16) |  | 57,3±13,3 |  |
|  | TAM | 4.10^6^ | 3 | | 3 (16 ;16 ;18) | ns | 56,2±12 |  |
|  | So | 1.10^4^ | 3 | | 3 (21 ;21 ;24) |  | 74,8±2,2 |  |
|  | TAM | 1.10^4^ | 3 | | 3 (21 ;23 ;23) | ns | 69,78±9,9 |  |
|  | So | 1.10^3^ | 3 | | 3 (25 ;25 ;25) |  | 64,8±13,3 |  |
|  | TAM | 1.10^3^ | 3 | | 3 (25 ;28 ;28) | ns | 79,5±1,8 |  |
|  | So | 1.10^2^ | 3 | | 3 (30 ;32 ;36) |  | 79,3±0,8 |  |
|  | TAM | 1.10^2^ | 3 | | 3 (30 ;34 ;36) | ns | 75,6±1,8 |  |
