# Supplementary material for: NFAT transcription factors are essential and redundant actors for leukemia initiating potential in T-cell acute lymphoblastic leukemia
Source: PLoS One. 2021 Jul 7;16(7):e0254184. doi: 10.1371/journal.pone.0254184 (PMC8263285; doi:10.1371/journal.pone.0254184)
Supplement: S2 Table — (DOCX) [file pone.0254184.s009.docx]

**S2 Table. NFAT-dependent transcriptome in T-ALL**

**Up-regulated probe sets upon NFAT inactivation**

| Gene Symbol | Gene Name | Fold-Change | P-Value |
| --- | --- | --- | --- |
| Gimap4 | GTPase, IMAP family member 4 | 4,20 | 3,71E-02 |
| Gimap4 | GTPase, IMAP family member 4 | 4,18 | 2,60E-02 |
| Pou2af1 | POU domain, class 2, associating factor 1 | 4,10 | 3,42E-02 |
| Btla | B and T lymphocyte associated | 4,07 | 2,20E-03 |
| Cd300lf | CD300 antigen like family member F | 3,63 | 3,02E-02 |
| 5830411N06Rik | RIKEN cDNA 5830411N06 gene | 3,63 | 1,17E-03 |
| Cdyl2 | chromodomain protein, Y chromosome-like 2 | 3,38 | 1,08E-02 |
| Fam183b | family with sequence similarity 183, member B | 3,27 | 2,40E-02 |
| Gimap7 | GTPase, IMAP family member 7 | 3,23 | 1,97E-02 |
| Cd163l1 | CD163 molecule-like 1 | 3,19 | 1,13E-02 |
| 2810459M11Rik | RIKEN cDNA 2810459M11 gene | 3,18 | 1,54E-03 |
| Bex6 | brain expressed gene 6 | 3,15 | 2,11E-02 |
| St6gal1 | beta galactoside alpha 2,6 sialyltransferase 1 | 3,06 | 3,40E-02 |
| Stc1 | stanniocalcin 1 | 2,88 | 1,12E-02 |
| Hemgn | hemogen | 2,80 | 3,00E-02 |
| Cdkn1a | cyclin-dependent kinase inhibitor 1A (P21) | 2,74 | 1,30E-02 |
| Irf4 | interferon regulatory factor 4 | 2,73 | 1,57E-03 |
| Cdkn1a | cyclin-dependent kinase inhibitor 1A (P21) | 2,73 | 4,03E-02 |
| Gjb2 | gap junction protein, beta 2 | 2,68 | 6,48E-03 |
| Cdyl2 | Chromodomain protein, Y chromosome-like 2 | 2,66 | 2,70E-02 |
| Plac8 | placenta-specific 8 | 2,65 | 4,38E-02 |
| Ndrg1 | N-myc downstream regulated gene 1 | 2,64 | 2,24E-02 |
| Upb1 | ureidopropionase, beta | 2,62 | 9,00E-03 |
| 2010007H06Rik | RIKEN cDNA 2010007H06 gene | 2,62 | 6,98E-03 |
| Ndrg1 | N-myc downstream regulated gene 1 | 2,61 | 4,65E-02 |
| Ndrg1 | N-myc downstream regulated gene 1 | 2,60 | 4,72E-02 |
| Ppm1l | protein phosphatase 1 (formerly 2C)-like | 2,57 | 2,09E-02 |
| Abcb1b | ATP-binding cassette, sub-family B (MDR/TAP), member 1B | 2,54 | 3,29E-03 |
| Nr4a1 | nuclear receptor subfamily 4, group A, member 1 | 2,53 | 2,31E-02 |
| Ndrg1 | N-myc downstream regulated gene 1 | 2,48 | 2,37E-02 |
| Ugcg | UDP-glucose ceramide glucosyltransferase | 2,39 | 3,45E-03 |
| Mboat1 | membrane bound O-acyltransferase domain containing 1 | 2,37 | 1,91E-02 |
| Ugcg | UDP-glucose ceramide glucosyltransferase | 2,35 | 2,17E-02 |
| Spats2 | spermatogenesis associated, serine-rich 2 | 2,35 | 4,89E-02 |
| Ampd1 | adenosine monophosphate deaminase 1 | 2,32 | 1,62E-02 |
| Chsy1 | chondroitin sulfate synthase 1 | 2,32 | 6,25E-03 |
| Gadd45b | growth arrest and DNA-damage-inducible 45 beta | 2,30 | 6,48E-03 |
| Coro2a | coronin, actin binding protein 2A | 2,30 | 2,23E-02 |
| Gadd45b | growth arrest and DNA-damage-inducible 45 beta | 2,27 | 4,39E-03 |
| AW061096 | expressed sequence AW061096 | 2,26 | 3,37E-05 |
| Spin2 | spindlin family, member 2 | 2,18 | 2,14E-02 |
| Runx3 | runt related transcription factor 3 | 2,17 | 4,03E-02 |
| Igf1r | insulin-like growth factor I receptor | 2,17 | 1,15E-03 |
| D630039A03Rik | RIKEN cDNA D630039A03 gene | 2,16 | 2,68E-02 |
| Itih5 | inter-alpha (globulin) inhibitor H5 | 2,12 | 8,83E-03 |
| Ms4a4c | membrane-spanning 4-domains, subfamily A, member 4C | 2,12 | 3,88E-02 |
| Ppm1l | protein phosphatase 1 (formerly 2C)-like | 2,10 | 1,32E-02 |
| Spib | Spi-B transcription factor (Spi-1/PU.1 related) | 2,09 | 1,51E-02 |
| Kmo | kynurenine 3-monooxygenase (kynurenine 3-hydroxylase) | 2,06 | 3,93E-02 |
| Erbb3 | v-erb-b2 erythroblastic leukemia viral oncogene homolog 3 (avian) | 2,02 | 2,82E-02 |
| Cobll1 | Cobl-like 1 | 1,98 | 4,04E-02 |
| Relb | avian reticuloendotheliosis viral (v-rel) oncogene related B | 1,98 | 1,50E-02 |
| Dusp5 | dual specificity phosphatase 5 | 1,97 | 1,86E-03 |
| Egln3 | EGL nine homolog 3 (C. elegans) | 1,95 | 6,92E-03 |
| Nab1 | Ngfi-A binding protein 1 | 1,95 | 2,54E-02 |
| Ly6a | lymphocyte antigen 6 complex, locus A | 1,91 | 3,77E-02 |
| Bcl3 | B-cell leukemia/lymphoma 3 | 1,91 | 4,72E-02 |
| Tpm4 | tropomyosin 4 | 1,91 | 4,29E-02 |
| Klf3 | Kruppel-like factor 3 (basic) | 1,90 | 3,73E-02 |
| Stom | stomatin | 1,89 | 2,83E-02 |
| Sdc1 | syndecan 1 | 1,88 | 2,16E-02 |
| Sdc1 | syndecan 1 | 1,86 | 3,42E-02 |
| Ccdc141 | coiled-coil domain containing 141 | 1,85 | 2,41E-02 |
| D18Ertd653e | DNA segment, Chr 18, ERATO Doi 653, expressed | 1,84 | 4,15E-02 |
| Cnn3 | calponin 3, acidic | 1,84 | 1,87E-02 |
| Ms4a6d | membrane-spanning 4-domains, subfamily A, member 6D | 1,83 | 4,43E-02 |
| Abcb1a | ATP-binding cassette, sub-family B (MDR/TAP), member 1A | 1,83 | 4,26E-02 |
| Tfrc | transferrin receptor | 1,83 | 2,48E-02 |
| Frmd4b | FERM domain containing 4B | 1,81 | 4,68E-02 |
| Stom | stomatin | 1,81 | 2,56E-02 |
| Ddn | dendrin | 1,81 | 4,64E-03 |
| 2510009E07Rik | RIKEN cDNA 2510009E07 gene | 1,81 | 1,21E-03 |
| Cd24a | CD24a antigen | 1,80 | 4,24E-02 |
| P2rx7 | purinergic receptor P2X, ligand-gated ion channel, 7 | 1,80 | 1,98E-02 |
| 2010007H06Rik | RIKEN cDNA 2010007H06 gene | 1,78 | 1,15E-02 |
| P2rx7 | purinergic receptor P2X, ligand-gated ion channel, 7 | 1,77 | 3,24E-03 |
| Chchd10 | coiled-coil-helix-coiled-coil-helix domain containing 10 | 1,77 | 1,63E-02 |
| Itih5 | inter-alpha (globulin) inhibitor H5 | 1,76 | 1,99E-04 |
| Chchd10 | coiled-coil-helix-coiled-coil-helix domain containing 10 | 1,76 | 3,30E-03 |
| Psrc1 | proline/serine-rich coiled-coil 1 | 1,76 | 1,48E-02 |
| Epsti1 | epithelial stromal interaction 1 (breast) | 1,76 | 2,69E-02 |
| Itih5 | inter-alpha (globulin) inhibitor H5 | 1,75 | 5,46E-03 |
| Sdc1 | syndecan 1 | 1,75 | 7,02E-03 |
| Gpr65 | G-protein coupled receptor 65 | 1,74 | 2,52E-02 |
| Cdk5r1 | cyclin-dependent kinase 5, regulatory subunit 1 (p35) | 1,74 | 4,44E-02 |
| Grn | granulin | 1,74 | 1,99E-02 |
| Cnn3 | calponin 3, acidic | 1,73 | 9,87E-03 |
| Cdk5r1 | cyclin-dependent kinase 5, regulatory subunit 1 (p35) | 1,73 | 8,73E-03 |
| Jakmip1 | janus kinase and microtubule interacting protein 1 | 1,72 | 2,84E-02 |
| Tnfsf10 | tumor necrosis factor (ligand) superfamily, member 10 | 1,72 | 4,62E-02 |
| 6530402F18Rik | RIKEN cDNA 6530402F18 gene | 1,72 | 1,72E-02 |
| Nrarp | Notch-regulated ankyrin repeat protein | 1,71 | 3,11E-02 |
| Pcyt1a | phosphate cytidylyltransferase 1, choline, alpha isoform | 1,70 | 2,70E-02 |
| Grn | granulin | 1,70 | 4,43E-02 |
| Fkbp1a | FK506 binding protein 1a | 1,69 | 2,55E-02 |
| Cnn3 | calponin 3, acidic | 1,68 | 1,24E-02 |
| Dr1 | down-regulator of transcription 1 | 1,68 | 7,77E-03 |
| Ugcg | UDP-glucose ceramide glucosyltransferase | 1,68 | 1,90E-02 |
| Atl3 | atlastin GTPase 3 | 1,66 | 3,37E-02 |
| Serp1 | stress-associated endoplasmic reticulum protein 1 | 1,65 | 7,91E-03 |
| Abce1 | ATP-binding cassette, sub-family E (OABP), member 1 | 1,64 | 3,33E-02 |
| Ehd3 | EH-domain containing 3 | 1,64 | 2,70E-02 |
| Hopx | HOP homeobox | 1,64 | 9,88E-04 |
| Bst2 | bone marrow stromal cell antigen 2 | 1,64 | 1,09E-02 |
| Lpar4 | lysophosphatidic acid receptor 4 | 1,63 | 3,22E-02 |
| Gpr25 | G protein-coupled receptor 25 | 1,62 | 4,60E-02 |
| Spred1 | sprouty protein with EVH-1 domain 1, related sequence | 1,62 | 3,40E-04 |
| --- | --- | 1,61 | 4,55E-02 |
| Bdh1 | 3-hydroxybutyrate dehydrogenase, type 1 | 1,61 | 4,50E-02 |
| Ptp4a3 | protein tyrosine phosphatase 4a3 | 1,60 | 6,07E-03 |
| Plekha1 | pleckstrin homology domain containing, family A (phosphoinositide binding specific) member 1 | 1,59 | 2,04E-03 |
| Sdccag3 | serologically defined colon cancer antigen 3 | 1,58 | 3,77E-02 |
| Stard3nl | STARD3 N-terminal like | 1,58 | 3,83E-02 |
| Camk4 | calcium/calmodulin-dependent protein kinase IV | 1,57 | 3,53E-02 |
| Rpe | ribulose-5-phosphate-3-epimerase | 1,57 | 3,49E-02 |
| Pdha1 | pyruvate dehydrogenase E1 alpha 1 | 1,56 | 2,43E-02 |
| Als2cr12 | amyotrophic lateral sclerosis 2 (juvenile) chromosome region, candidate 12 (human) | 1,56 | 7,43E-03 |
| Mgat5 | mannoside acetylglucosaminyltransferase 5 | 1,56 | 2,15E-03 |
| Tg | thyroglobulin | 1,56 | 4,55E-02 |
| Ms4a6d | membrane-spanning 4-domains, subfamily A, member 6D | 1,56 | 1,75E-02 |
| Slc11a2 | solute carrier family 11 (proton-coupled divalent metal ion transporters), member 2 | 1,56 | 3,75E-02 |
| Dap | death-associated protein | 1,56 | 3,12E-02 |
| --- | --- | 1,55 | 9,80E-03 |
| Cdv3 | carnitine deficiency-associated gene expressed in ventricle 3 | 1,55 | 1,45E-02 |
| Api5 | apoptosis inhibitor 5 | 1,55 | 2,47E-02 |
| Bpnt1 | bisphosphate 3'-nucleotidase 1 | 1,55 | 3,45E-02 |
| Parp14 | poly (ADP-ribose) polymerase family, member 14 | 1,54 | 1,79E-02 |
| Hspa13 | heat shock protein 70 family, member 13 | 1,54 | 2,17E-02 |
| AI447881 | expressed sequence AI447881 | 1,53 | 1,88E-04 |
| Nampt | nicotinamide phosphoribosyltransferase | 1,53 | 3,89E-02 |
| Zfp428 | zinc finger protein 428 | 1,53 | 1,94E-02 |
| Amd1 | S-adenosylmethionine decarboxylase 1 | 1,53 | 4,09E-02 |
| Ier2 | immediate early response 2 | 1,53 | 3,21E-02 |
| Prkca | protein kinase C, alpha | 1,53 | 4,76E-02 |
| Orc1 | origin recognition complex, subunit 1 | 1,53 | 3,93E-02 |
| Endod1 | endonuclease domain containing 1 | 1,52 | 3,43E-02 |
| Iqgap2 | IQ motif containing GTPase activating protein 2 | 1,51 | 3,04E-02 |
| Ccdc102a | coiled-coil domain containing 102A | 1,50 | 7,77E-03 |

**Down-regulated probe sets upon NFAT inactivation**

| Gene Symbol | Gene Name | Fold-Change | P-Value |
| --- | --- | --- | --- |
| Lamb1 | laminin B1 | 4,54 | 1,27E-02 |
| Vcan | versican | 4,31 | 1,02E-02 |
| Tcrg-V4 | T-cell receptor gamma, variable 4 | 3,99 | 1,11E-03 |
| 1110036O03Rik | RIKEN cDNA 1110036O03 gene | 3,91 | 3,44E-02 |
| Tcrg-V4 | T-cell receptor gamma, variable 4 | 3,70 | 8,23E-04 |
| Tcrg-V4 | T-cell receptor gamma, variable 4 | 3,50 | 1,91E-02 |
| Mpp4 | membrane protein, palmitoylated 4 (MAGUK p55 subfamily member 4) | 3,42 | 1,62E-02 |
| Arrdc3 | arrestin domain containing 3 | 3,40 | 4,93E-02 |
| Ephx1 | epoxide hydrolase 1, microsomal | 2,97 | 1,58E-02 |
| Gm14446 | predicted gene 14446 | 2,88 | 1,24E-02 |
| Samd9l | sterile alpha motif domain containing 9-like | 2,80 | 4,22E-02 |
| Nebl | nebulette | 2,69 | 2,60E-02 |
| 9530077C05Rik | RIKEN cDNA 9530077C05 gene | 2,69 | 6,21E-04 |
| Tns3 | tensin 3 | 2,63 | 2,34E-02 |
| Arl4a | ADP-ribosylation factor-like 4A | 2,55 | 1,14E-02 |
| 4632428N05Rik | RIKEN cDNA 4632428N05 gene | 2,52 | 4,26E-02 |
| --- | --- | 2,49 | 3,28E-02 |
| Lax1 | lymphocyte transmembrane adaptor 1 | 2,48 | 4,04E-02 |
| Evi5 | ecotropic viral integration site 5 | 2,45 | 3,61E-03 |
| Tox | thymocyte selection-associated high mobility group box | 2,44 | 4,19E-03 |
| Tox | thymocyte selection-associated high mobility group box | 2,40 | 7,91E-04 |
| Lamb1 | laminin B1 | 2,38 | 4,85E-02 |
| Frat2 | frequently rearranged in advanced T-cell lymphomas 2 | 2,36 | 1,13E-02 |
| Tcrg-V2 /// Tcrg-V3 | T-cell receptor gamma, variable 2 /// T-cell receptor gamma, variable 3 | 2,31 | 2,82E-02 |
| --- | --- | 2,30 | 1,32E-02 |
| 9530028C05 | hypothetical protein 9530028C05 | 2,28 | 2,19E-02 |
| 9530077C05Rik | RIKEN cDNA 9530077C05 gene | 2,28 | 5,79E-05 |
| Ifngr1 | interferon gamma receptor 1 | 2,23 | 1,53E-02 |
| Evi5 | ecotropic viral integration site 5 | 2,22 | 5,67E-03 |
| 4930550C14Rik | RIKEN cDNA 4930550C14 gene | 2,21 | 1,69E-02 |
| Ube2e2 | ubiquitin-conjugating enzyme E2E 2 (UBC4/5 homolog, yeast) | 2,18 | 1,89E-02 |
| Lmo4 | LIM domain only 4 | 2,17 | 3,93E-02 |
| Gbp6 | guanylate binding protein 6 | 2,15 | 2,57E-03 |
| Parm1 | prostate androgen-regulated mucin-like protein 1 | 2,14 | 4,84E-02 |
| Fhit | fragile histidine triad gene | 2,12 | 2,41E-03 |
| Ypel2 | yippee-like 2 (Drosophila) | 2,08 | 2,97E-03 |
| Carns1 | carnosine synthase 1 | 2,05 | 3,08E-02 |
| --- | --- | 2,03 | 3,28E-02 |
| Ahnak | AHNAK nucleoprotein (desmoyokin) | 2,03 | 6,87E-03 |
| BB165335 | expressed sequence BB165335 | 2,01 | 4,52E-02 |
| Usp3 | ubiquitin specific peptidase 3 | 2,01 | 2,95E-02 |
| Fxyd5 | FXYD domain-containing ion transport regulator 5 | 1,99 | 1,02E-03 |
| Spsb4 | splA/ryanodine receptor domain and SOCS box containing 4 | 1,98 | 2,54E-02 |
| Cass4 | Cas scaffolding protein family member 4 | 1,96 | 4,39E-02 |
| Gbp6 | guanylate binding protein 6 | 1,95 | 1,75E-02 |
| AW050198 | expressed sequence AW050198 | 1,94 | 3,69E-02 |
| Nr3c2 | nuclear receptor subfamily 3, group C, member 2 | 1,93 | 4,42E-02 |
| Ypel2 | yippee-like 2 (Drosophila) | 1,93 | 1,68E-02 |
| Idh3a | isocitrate dehydrogenase 3 (NAD+) alpha | 1,91 | 1,49E-02 |
| Plcxd2 | phosphatidylinositol-specific phospholipase C, X domain containing 2 | 1,90 | 1,32E-02 |
| Themis | Thymocyte selection associated | 1,89 | 4,38E-02 |
| Hspa4l | heat shock protein 4 like | 1,88 | 4,00E-03 |
| Usp3 | ubiquitin specific peptidase 3 | 1,88 | 4,68E-02 |
| Gjc1 | gap junction protein, gamma 1 | 1,88 | 6,54E-03 |
| Aldh6a1 | aldehyde dehydrogenase family 6, subfamily A1 | 1,87 | 3,92E-02 |
| LOC545086 | hypothetical protein LOC545086 | 1,87 | 9,15E-03 |
| Slc45a3 | solute carrier family 45, member 3 | 1,87 | 1,31E-02 |
| Prkch | protein kinase C, eta | 1,86 | 1,56E-03 |
| Pkig | protein kinase inhibitor, gamma | 1,85 | 2,73E-02 |
| Mtss1 | metastasis suppressor 1 | 1,85 | 6,31E-03 |
| Cdh22 | cadherin 22 | 1,85 | 4,24E-02 |
| --- | --- | 1,84 | 1,61E-02 |
| Rgs2 | regulator of G-protein signaling 2 | 1,84 | 2,58E-04 |
| AI661384 | expressed sequence AI661384 | 1,83 | 3,00E-02 |
| D1Ertd564e | DNA segment, Chr 1, ERATO Doi 564, expressed | 1,82 | 6,99E-03 |
| --- | --- | 1,81 | 2,28E-02 |
| --- | --- | 1,81 | 1,46E-02 |
| Treml2 | triggering receptor expressed on myeloid cells-like 2 | 1,81 | 1,49E-02 |
| --- | --- | 1,80 | 2,76E-02 |
| --- | --- | 1,80 | 1,77E-02 |
| Ikzf3 | IKAROS family zinc finger 3 | 1,79 | 4,52E-02 |
| Hspa4l | heat shock protein 4 like | 1,79 | 2,00E-02 |
| --- | --- | 1,78 | 4,31E-02 |
| Dnajb4 | DnaJ (Hsp40) homolog, subfamily B, member 4 | 1,78 | 9,36E-04 |
| E230032D23Rik | RIKEN cDNA E230032D23 gene | 1,77 | 1,11E-03 |
| Mtss1 | metastasis suppressor 1 | 1,77 | 1,40E-02 |
| Ampd3 | adenosine monophosphate deaminase 3 | 1,76 | 4,83E-02 |
| Tpp1 | tripeptidyl peptidase I | 1,76 | 2,87E-02 |
| Sgk1 | serum/glucocorticoid regulated kinase 1 | 1,75 | 3,68E-02 |
| --- | --- | 1,75 | 3,80E-02 |
| Tmem218 | transmembrane protein 218 | 1,75 | 2,20E-02 |
| Gmfg | glia maturation factor, gamma | 1,75 | 4,87E-02 |
| --- | --- | 1,75 | 4,17E-02 |
| Nedd4 | neural precursor cell expressed, developmentally down-regulated 4 | 1,75 | 1,97E-02 |
| Tpcn1 | two pore channel 1 | 1,75 | 4,20E-03 |
| E330018D03Rik | RIKEN cDNA E330018D03 gene | 1,74 | 4,11E-02 |
| Gm5914 | predicted gene 5914 | 1,74 | 3,13E-02 |
| Sesn1 | sestrin 1 | 1,74 | 1,84E-02 |
| Sesn1 | sestrin 1 | 1,73 | 2,85E-02 |
| 9530077C05Rik | RIKEN cDNA 9530077C05 gene | 1,73 | 2,04E-02 |
| --- | --- | 1,73 | 3,77E-02 |
| Sesn1 | sestrin 1 | 1,72 | 2,86E-02 |
| Spata6 | spermatogenesis associated 6 | 1,72 | 1,26E-02 |
| H2-Q2 | histocompatibility 2, Q region locus 2 | 1,71 | 8,79E-03 |
| Slc38a9 | solute carrier family 38, member 9 | 1,71 | 4,29E-02 |
| 4921509J17Rik /// Hspa4l | RIKEN cDNA 4921509J17 gene /// heat shock protein 4 like | 1,71 | 3,65E-02 |
| 4930578N16Rik | RIKEN cDNA 4930578N16 gene | 1,71 | 9,29E-03 |
| Ramp1 | receptor (calcitonin) activity modifying protein 1 | 1,70 | 1,36E-02 |
| Gch1 | GTP cyclohydrolase 1 | 1,70 | 1,75E-03 |
| Pde4dip | phosphodiesterase 4D interacting protein (myomegalin) | 1,70 | 3,03E-02 |
| Plcxd2 | phosphatidylinositol-specific phospholipase C, X domain containing 2 | 1,70 | 6,54E-03 |
| Ncoa1 | nuclear receptor coactivator 1 | 1,69 | 1,32E-02 |
| AI426330 | expressed sequence AI426330 | 1,69 | 2,19E-02 |
| Sacm1l | SAC1 (suppressor of actin mutations 1, homolog)-like (S. cerevisiae) | 1,69 | 4,49E-02 |
| Bzrap1 | benzodiazapine receptor associated protein 1 | 1,68 | 3,89E-03 |
| Spsb4 | splA/ryanodine receptor domain and SOCS box containing 4 | 1,68 | 4,13E-02 |
| --- | --- | 1,68 | 3,20E-02 |
| Csrnp1 | cysteine-serine-rich nuclear protein 1 | 1,67 | 2,09E-02 |
| 9630013D21Rik | RIKEN cDNA 9630013D21 gene | 1,67 | 1,03E-02 |
| Amn1 | Antagonist of mitotic exit network 1 homolog (S. cerevisiae) | 1,67 | 1,13E-02 |
| Cd84 | CD84 antigen | 1,67 | 2,60E-02 |
| Scai | suppressor of cancer cell invasion | 1,67 | 5,00E-03 |
| Rilpl2 | Rab interacting lysosomal protein-like 2 | 1,67 | 2,70E-03 |
| Ddhd2 | DDHD domain containing 2 | 1,66 | 6,23E-03 |
| Slc12a6 | solute carrier family 12, member 6 | 1,66 | 2,27E-02 |
| Trip4 | thyroid hormone receptor interactor 4 | 1,66 | 4,05E-02 |
| 2010004M13Rik | RIKEN cDNA 2010004M13 gene | 1,66 | 3,36E-02 |
| Foxp1 | Forkhead box P1 | 1,66 | 3,97E-02 |
| Ldlrap1 | low density lipoprotein receptor adaptor protein 1 | 1,66 | 3,46E-02 |
| 3110057O12Rik /// Gm2011 | RIKEN cDNA 3110057O12 gene /// predicted gene 2011 | 1,65 | 3,39E-02 |
| Dnahc8 | dynein, axonemal, heavy chain 8 | 1,64 | 2,08E-02 |
| --- | --- | 1,64 | 1,81E-02 |
| Atp5c1 | ATP synthase, H+ transporting, mitochondrial F1 complex, gamma polypeptide 1 | 1,64 | 4,40E-02 |
| Tmem19 | Transmembrane protein 19 | 1,64 | 4,60E-02 |
| Ncoa1 | nuclear receptor coactivator 1 | 1,64 | 1,58E-02 |
| 2900056M20Rik | RIKEN cDNA 2900056M20 gene | 1,63 | 4,32E-02 |
| Phf6 | PHD finger protein 6 | 1,63 | 2,20E-03 |
| Pcmtd2 | protein-L-isoaspartate (D-aspartate) O-methyltransferase domain containing 2 | 1,62 | 4,13E-02 |
| Setd4 | SET domain containing 4 | 1,62 | 4,25E-02 |
| Arpp21 | cyclic AMP-regulated phosphoprotein, 21 | 1,62 | 1,33E-02 |
| Satb1 | special AT-rich sequence binding protein 1 | 1,61 | 3,96E-02 |
| --- | --- | 1,61 | 3,14E-02 |
| Lman2l | lectin, mannose-binding 2-like | 1,61 | 2,90E-02 |
| Cfl2 | cofilin 2, muscle | 1,61 | 1,87E-02 |
| Pnrc1 | proline-rich nuclear receptor coactivator 1 | 1,61 | 3,23E-02 |
| Gse1 | genetic suppressor element 1 | 1,61 | 1,67E-02 |
| Gtdc1 | glycosyltransferase-like domain containing 1 | 1,60 | 4,90E-02 |
| Ramp1 | receptor (calcitonin) activity modifying protein 1 | 1,60 | 1,84E-03 |
| Fam199x | family with sequence similarity 199, X-linked | 1,60 | 1,97E-02 |
| --- | --- | 1,60 | 2,05E-02 |
| Pyhin1 | pyrin and HIN domain family, member 1 | 1,60 | 2,65E-02 |
| Col27a1 | collagen, type XXVII, alpha 1 | 1,60 | 4,29E-02 |
| Ppp1r3b | protein phosphatase 1, regulatory (inhibitor) subunit 3B | 1,60 | 3,79E-02 |
| Nedd4 | neural precursor cell expressed, developmentally down-regulated 4 | 1,59 | 1,17E-02 |
| Tube1 | epsilon-tubulin 1 | 1,59 | 4,04E-02 |
| Vamp4 | vesicle-associated membrane protein 4 | 1,59 | 6,33E-03 |
| Pkig | protein kinase inhibitor, gamma | 1,59 | 2,60E-02 |
| Stat5b | signal transducer and activator of transcription 5B | 1,59 | 3,41E-02 |
| Slc12a6 | solute carrier family 12, member 6 | 1,59 | 2,43E-02 |
| Hspa4l | heat shock protein 4 like | 1,59 | 4,88E-02 |
| Nudt16 | nudix (nucleoside diphosphate linked moiety X)-type motif 16 | 1,58 | 2,84E-02 |
| Dedd2 | death effector domain-containing DNA binding protein 2 | 1,58 | 4,42E-03 |
| Tmbim1 | transmembrane BAX inhibitor motif containing 1 | 1,58 | 2,12E-02 |
| Slc44a2 | solute carrier family 44, member 2 | 1,58 | 2,98E-02 |
| Fam199x | family with sequence similarity 199, X-linked | 1,58 | 1,39E-03 |
| --- | --- | 1,57 | 2,64E-02 |
| Parp16 | poly (ADP-ribose) polymerase family, member 16 | 1,57 | 6,99E-03 |
| Arrb1 | arrestin, beta 1 | 1,57 | 2,79E-02 |
| Col27a1 | collagen, type XXVII, alpha 1 | 1,57 | 4,09E-02 |
| Naip6 | NLR family, apoptosis inhibitory protein 6 | 1,57 | 2,56E-02 |
| Fut8 | fucosyltransferase 8 | 1,57 | 1,35E-02 |
| Acp5 | acid phosphatase 5, tartrate resistant | 1,57 | 7,14E-03 |
| Skiv2l2 | superkiller viralicidic activity 2-like 2 (S. cerevisiae) | 1,56 | 1,84E-02 |
| A630033H20Rik | RIKEN cDNA A630033H20 gene | 1,56 | 2,13E-02 |
| Rcbtb2 | regulator of chromosome condensation (RCC1) and BTB (POZ) domain containing protein 2 | 1,56 | 2,42E-03 |
| Lman2l | lectin, mannose-binding 2-like | 1,56 | 1,72E-02 |
| LOC100044751 | hypothetical LOC100044751 | 1,56 | 4,93E-02 |
| Hist2h2be | histone cluster 2, H2be | 1,56 | 2,25E-02 |
| Rdh10 | retinol dehydrogenase 10 (all-trans) | 1,56 | 4,05E-02 |
| Rere | arginine glutamic acid dipeptide (RE) repeats | 1,55 | 2,78E-02 |
| Sfrs18 | serine/arginine-rich splicing factor 18 | 1,55 | 2,88E-02 |
| Atp2a3 | ATPase, Ca++ transporting, ubiquitous | 1,55 | 1,40E-02 |
| Mbtd1 | mbt domain containing 1 | 1,55 | 1,13E-02 |
| Mthfd2l | methylenetetrahydrofolate dehydrogenase (NADP+ dependent) 2-like | 1,55 | 1,43E-02 |
| Atg12 | autophagy-related 12 (yeast) | 1,54 | 7,23E-03 |
| Abhd8 | abhydrolase domain containing 8 | 1,54 | 1,89E-02 |
| Itpkb | inositol 1,4,5-trisphosphate 3-kinase B | 1,54 | 4,09E-02 |
| Slc45a3 | solute carrier family 45, member 3 | 1,54 | 3,89E-02 |
| Bach1 | BTB and CNC homology 1 | 1,54 | 1,48E-03 |
| --- | --- | 1,54 | 3,55E-02 |
| Slc44a2 | solute carrier family 44, member 2 | 1,54 | 2,11E-02 |
| Zfp182 | zinc finger protein 182 | 1,53 | 8,44E-03 |
| Fam53b | family with sequence similarity 53, member B | 1,53 | 2,16E-02 |
| Pdk1 | pyruvate dehydrogenase kinase, isoenzyme 1 | 1,53 | 3,59E-03 |
| LOC620419 | zinc finger protein 669-like | 1,53 | 2,89E-02 |
| Pdk1 | pyruvate dehydrogenase kinase, isoenzyme 1 | 1,52 | 1,35E-02 |
| Cd96 | CD96 antigen | 1,52 | 1,45E-05 |
| Pip4k2a | phosphatidylinositol-5-phosphate 4-kinase, type II, alpha | 1,52 | 5,73E-03 |
| D230044B12Rik | RIKEN cDNA D230044B12 gene | 1,52 | 3,06E-02 |
| Lyst | lysosomal trafficking regulator | 1,52 | 2,67E-02 |
| Sfrs18 | serine/arginine-rich splicing factor 18 | 1,52 | 3,39E-02 |
| LOC100502594 | hypothetical LOC100502594 | 1,52 | 4,07E-02 |
| Prex1 | phosphatidylinositol-3,4,5-trisphosphate-dependent Rac exchange factor 1 | 1,51 | 2,74E-02 |
| Xpr1 | xenotropic and polytropic retrovirus receptor 1 | 1,51 | 4,64E-02 |
| 1700097N02Rik | RIKEN cDNA 1700097N02 gene | 1,51 | 2,27E-02 |
| --- | --- | 1,51 | 3,27E-02 |
| Akap8l | A kinase (PRKA) anchor protein 8-like | 1,51 | 1,97E-04 |
| Dpp4 | dipeptidylpeptidase 4 | 1,51 | 1,19E-02 |
| --- | --- | 1,51 | 1,58E-02 |
| Cxxc5 | CXXC finger 5 | 1,51 | 1,42E-02 |
| Map4k3 | mitogen-activated protein kinase kinase kinase kinase 3 | 1,51 | 2,98E-02 |
| Tbc1d14 | TBC1 domain family, member 14 | 1,50 | 1,74E-02 |
| Otos | otospiralin | 1,50 | 4,84E-02 |
| Atr | Ataxia telangiectasia and Rad3 related | 1,50 | 3,43E-02 |
| --- | --- | 1,50 | 4,65E-02 |
