# Supplementary material for: NFAT transcription factors are essential and redundant actors for leukemia initiating potential in T-cell acute lymphoblastic leukemia
Source: PLoS One. 2021 Jul 7;16(7):e0254184. doi: 10.1371/journal.pone.0254184 (PMC8263285; doi:10.1371/journal.pone.0254184)
Supplement: S3 Table — (DOCX) [file pone.0254184.s010.docx]

**S3 Table. Overlap between NFAT- and Calcineurin-dependent transcriptomes**

**Common up-regulated probe sets in NFAT or CnB inactivated T-ALL cells**

NFAT CnB

| Gene Symbol | Gene Name | Fold-Change | P-Value | Fold-Change | P-Value | P-Value |
| --- | --- | --- | --- | --- | --- | --- |
| Pou2af1 | POU domain, class 2, associating factor 1 | 4,10 | 3,42E-02 | 1,75 | 2,29E-02 | 2,29E-02 |
| 5830411N06Rik | RIKEN cDNA 5830411N06 gene | 3,63 | 1,17E-03 | 1,86 | 3,93E-03 | 3,93E-03 |
| Cdyl2 | chromodomain protein, Y chromosome-like 2 | 3,38 | 1,08E-02 | 1,97 | 1,72E-03 | 1,72E-03 |
| Fam183b | family with sequence similarity 183, member B | 3,27 | 2,40E-02 | 2,73 | 4,72E-02 | 4,72E-02 |
| Gimap7 | GTPase, IMAP family member 7 | 3,23 | 1,97E-02 | 2,50 | 1,52E-02 | 1,52E-02 |
| Cd163l1 | CD163 molecule-like 1 | 3,19 | 1,13E-02 | 1,88 | 4,62E-02 | 4,62E-02 |
| 2810459M11Rik | RIKEN cDNA 2810459M11 gene | 3,18 | 1,54E-03 | 2,24 | 1,34E-03 | 1,34E-03 |
| Bex6 | brain expressed gene 6 | 3,15 | 2,11E-02 | 1,73 | 2,31E-03 | 2,31E-03 |
| Stc1 | stanniocalcin 1 | 2,88 | 1,12E-02 | 2,15 | 3,04E-02 | 3,04E-02 |
| Cdkn1a | cyclin-dependent kinase inhibitor 1A (P21) | 2,74 | 1,30E-02 | 2,49 | 2,48E-03 | 2,48E-03 |
| Cdkn1a | cyclin-dependent kinase inhibitor 1A (P21) | 2,73 | 4,03E-02 | 3,45 | 4,21E-04 | 4,21E-04 |
| Irf4 | interferon regulatory factor 4 | 2,73 | 1,57E-03 | 1,89 | 2,60E-05 | 2,60E-05 |
| Gjb2 | gap junction protein, beta 2 | 2,68 | 6,48E-03 | 5,16 | 4,44E-04 | 4,44E-04 |
| Cdyl2 | Chromodomain protein, Y chromosome-like 2 | 2,66 | 2,70E-02 | 2,31 | 1,24E-03 | 1,24E-03 |
| Abcb1b | ATP-binding cassette, sub-family B (MDR/TAP), member 1B | 2,54 | 3,29E-03 | 1,71 | 1,07E-02 | 1,07E-02 |
| Nr4a1 | nuclear receptor subfamily 4, group A, member 1 | 2,53 | 2,31E-02 | 5,64 | 8,89E-04 | 8,89E-04 |
| Ugcg | UDP-glucose ceramide glucosyltransferase | 2,39 | 3,45E-03 | 1,86 | 3,15E-03 | 3,15E-03 |
| Ugcg | UDP-glucose ceramide glucosyltransferase | 2,35 | 2,17E-02 | 1,87 | 2,53E-03 | 2,53E-03 |
| Chsy1 | chondroitin sulfate synthase 1 | 2,32 | 6,25E-03 | 1,96 | 4,76E-03 | 4,76E-03 |
| Spin2 | spindlin family, member 2 | 2,18 | 2,14E-02 | 1,60 | 2,63E-02 | 2,63E-02 |
| Igf1r | insulin-like growth factor I receptor | 2,17 | 1,15E-03 | 1,67 | 3,53E-02 | 3,53E-02 |
| Itih5 | inter-alpha (globulin) inhibitor H5 | 2,12 | 8,83E-03 | 2,52 | 3,57E-03 | 3,57E-03 |
| Ppm1l | protein phosphatase 1 (formerly 2C)-like | 2,10 | 1,32E-02 | 1,86 | 6,80E-04 | 6,80E-04 |
| Erbb3 | v-erb-b2 erythroblastic leukemia viral oncogene homolog 3 (avian) | 2,02 | 2,82E-02 | 3,47 | 6,16E-03 | 6,16E-03 |
| Cobll1 | Cobl-like 1 | 1,98 | 4,04E-02 | 1,55 | 4,47E-02 | 4,47E-02 |
| Relb | avian reticuloendotheliosis viral (v-rel) oncogene related B | 1,98 | 1,50E-02 | 1,63 | 1,09E-03 | 1,09E-03 |
| Dusp5 | dual specificity phosphatase 5 | 1,97 | 1,86E-03 | 2,54 | 2,98E-04 | 2,98E-04 |
| Egln3 | EGL nine homolog 3 (C. elegans) | 1,95 | 6,92E-03 | 1,72 | 8,21E-04 | 8,21E-04 |
| Ly6a | lymphocyte antigen 6 complex, locus A | 1,91 | 3,77E-02 | 2,34 | 8,96E-03 | 8,96E-03 |
| Klf3 | Kruppel-like factor 3 (basic) | 1,90 | 3,73E-02 | 1,71 | 1,74E-02 | 1,74E-02 |
| D18Ertd653e | DNA segment, Chr 18, ERATO Doi 653, expressed | 1,84 | 4,15E-02 | 2,16 | 4,27E-03 | 4,27E-03 |
| Ms4a6d | membrane-spanning 4-domains, subfamily A, member 6D | 1,83 | 4,43E-02 | 1,78 | 1,04E-02 | 1,04E-02 |
| Abcb1a | ATP-binding cassette, sub-family B (MDR/TAP), member 1A | 1,83 | 4,26E-02 | 3,20 | 1,72E-02 | 1,72E-02 |
| Ddn | dendrin | 1,81 | 4,64E-03 | 2,04 | 4,88E-04 | 4,88E-04 |
| 2510009E07Rik | RIKEN cDNA 2510009E07 gene | 1,81 | 1,21E-03 | 2,06 | 1,14E-03 | 1,14E-03 |
| Cd24a | CD24a antigen | 1,80 | 4,24E-02 | 1,61 | 1,12E-03 | 1,12E-03 |
| Chchd10 | coiled-coil-helix-coiled-coil-helix domain containing 10 | 1,77 | 1,63E-02 | 1,84 | 1,37E-03 | 1,37E-03 |
| Itih5 | inter-alpha (globulin) inhibitor H5 | 1,76 | 1,99E-04 | 2,65 | 5,28E-03 | 5,28E-03 |
| Chchd10 | coiled-coil-helix-coiled-coil-helix domain containing 10 | 1,76 | 3,30E-03 | 1,80 | 6,42E-03 | 6,42E-03 |
| Epsti1 | epithelial stromal interaction 1 (breast) | 1,76 | 2,69E-02 | 1,98 | 8,00E-04 | 8,00E-04 |
| Itih5 | inter-alpha (globulin) inhibitor H5 | 1,75 | 5,46E-03 | 2,05 | 1,23E-02 | 1,23E-02 |
| Jakmip1 | janus kinase and microtubule interacting protein 1 | 1,72 | 2,84E-02 | 1,72 | 3,37E-03 | 3,37E-03 |
| Ugcg | UDP-glucose ceramide glucosyltransferase | 1,68 | 1,90E-02 | 1,76 | 1,14E-03 | 1,14E-03 |
| Abce1 | ATP-binding cassette, sub-family E (OABP), member 1 | 1,64 | 3,33E-02 | 1,61 | 3,71E-02 | 3,71E-02 |
| Ehd3 | EH-domain containing 3 | 1,64 | 2,70E-02 | 1,93 | 9,71E-03 | 9,71E-03 |
| --- | --- | 1,61 | 4,55E-02 | 1,72 | 2,74E-02 | 2,74E-02 |
| Ptp4a3 | protein tyrosine phosphatase 4a3 | 1,60 | 6,07E-03 | 1,75 | 9,21E-03 | 9,21E-03 |
| Mgat5 | mannoside acetylglucosaminyltransferase 5 | 1,56 | 2,15E-03 | 2,00 | 1,77E-03 | 1,77E-03 |
| Ier2 | immediate early response 2 | 1,53 | 3,21E-02 | 1,56 | 1,55E-02 | 1,55E-02 |
| Iqgap2 | IQ motif containing GTPase activating protein 2 | 1,51 | 3,04E-02 | 1,55 | 3,29E-02 | 3,29E-02 |
| Ehd3 | EH-domain containing 3 | 1,50 | 1,52E-01 | 1,71 | 7,26E-03 | 7,26E-03 |

**Common down-regulated probe sets in NFAT or CnB inactivated T-ALL cells**

NFAT CnB

| Gene Symbol | Gene Name | Fold-Change | P-Value | Fold-Change | P-Value | P-Value |
| --- | --- | --- | --- | --- | --- | --- |
| Lamb1 | laminin B1 | 4,54 | 1,27E-02 | 2,98 | 4,65E-02 | 4,65E-02 |
| Tcrg-V4 | T-cell receptor gamma, variable 4 | 3,99 | 1,11E-03 | 2,31 | 1,80E-02 | 1,80E-02 |
| Tcrg-V4 | T-cell receptor gamma, variable 4 | 3,70 | 8,23E-04 | 2,22 | 2,01E-02 | 2,01E-02 |
| Tcrg-V4 | T-cell receptor gamma, variable 4 | 3,50 | 1,91E-02 | 2,69 | 1,74E-02 | 1,74E-02 |
| Ephx1 | epoxide hydrolase 1, microsomal | 2,97 | 1,58E-02 | 4,34 | 5,28E-04 | 5,28E-04 |
| Samd9l | sterile alpha motif domain containing 9-like | 2,80 | 4,22E-02 | 2,44 | 3,92E-02 | 3,92E-02 |
| 9530077C05Rik | RIKEN cDNA 9530077C05 gene | 2,69 | 6,21E-04 | 2,14 | 1,63E-02 | 1,63E-02 |
| Tns3 | tensin 3 | 2,63 | 2,34E-02 | 2,42 | 1,48E-02 | 1,48E-02 |
| Arl4a | ADP-ribosylation factor-like 4A | 2,55 | 1,14E-02 | 2,03 | 2,58E-02 | 2,58E-02 |
| Evi5 | ecotropic viral integration site 5 | 2,45 | 3,61E-03 | 2,25 | 1,49E-03 | 1,49E-03 |
| Tox | thymocyte selection-associated high mobility group box | 2,44 | 4,19E-03 | 2,03 | 1,08E-02 | 1,08E-02 |
| Tox | thymocyte selection-associated high mobility group box | 2,40 | 7,91E-04 | 1,96 | 1,22E-02 | 1,22E-02 |
| Frat2 | frequently rearranged in advanced T-cell lymphomas 2 | 2,36 | 1,13E-02 | 3,16 | 2,71E-03 | 2,71E-03 |
| Tcrg-V2 /// Tcrg-V3 | T-cell receptor gamma, variable 2 /// T-cell receptor gamma, variable 3 | 2,31 | 2,82E-02 | 2,26 | 2,37E-02 | 2,37E-02 |
| 9530077C05Rik | RIKEN cDNA 9530077C05 gene | 2,28 | 5,79E-05 | 2,24 | 1,63E-03 | 1,63E-03 |
| Evi5 | ecotropic viral integration site 5 | 2,22 | 5,67E-03 | 2,02 | 1,86E-03 | 1,86E-03 |
| Lmo4 | LIM domain only 4 | 2,17 | 3,93E-02 | 2,11 | 1,65E-02 | 1,65E-02 |
| Parm1 | prostate androgen-regulated mucin-like protein 1 | 2,14 | 4,84E-02 | 1,89 | 8,04E-03 | 8,04E-03 |
| Usp3 | ubiquitin specific peptidase 3 | 2,01 | 2,95E-02 | 1,52 | 4,31E-03 | 4,31E-03 |
| Hspa4l | heat shock protein 4 like | 1,88 | 4,00E-03 | 1,52 | 2,06E-02 | 2,06E-02 |
| Gjc1 | gap junction protein, gamma 1 | 1,88 | 6,54E-03 | 2,54 | 1,91E-03 | 1,91E-03 |
| Aldh6a1 | aldehyde dehydrogenase family 6, subfamily A1 | 1,87 | 3,92E-02 | 1,61 | 1,24E-02 | 1,24E-02 |
| Prkch | protein kinase C, eta | 1,86 | 1,56E-03 | 1,56 | 6,17E-03 | 6,17E-03 |
| Pkig | protein kinase inhibitor, gamma | 1,85 | 2,73E-02 | 1,59 | 5,56E-03 | 5,56E-03 |
| Ikzf3 | IKAROS family zinc finger 3 | 1,79 | 4,52E-02 | 1,68 | 7,44E-03 | 7,44E-03 |
| Hspa4l | heat shock protein 4 like | 1,79 | 2,00E-02 | 1,86 | 1,36E-02 | 1,36E-02 |
| Ampd3 | adenosine monophosphate deaminase 3 | 1,76 | 4,83E-02 | 1,65 | 2,35E-02 | 2,35E-02 |
| 9530077C05Rik | RIKEN cDNA 9530077C05 gene | 1,73 | 2,04E-02 | 2,26 | 3,82E-03 | 3,82E-03 |
| Spata6 | spermatogenesis associated 6 | 1,72 | 1,26E-02 | 1,55 | 3,13E-02 | 3,13E-02 |
| 4921509J17Rik /// Hspa4l | RIKEN cDNA 4921509J17 gene /// heat shock protein 4 like | 1,71 | 3,65E-02 | 1,75 | 1,71E-02 | 1,71E-02 |
| Tox | thymocyte selection-associated high mobility group box | 1,71 | 2,22E-01 | 1,99 | 8,15E-03 | 8,15E-03 |
| Rilpl2 | Rab interacting lysosomal protein-like 2 | 1,67 | 2,70E-03 | 1,92 | 3,71E-04 | 3,71E-04 |
| Ddhd2 | DDHD domain containing 2 | 1,66 | 6,23E-03 | 1,60 | 2,51E-02 | 2,51E-02 |
| Ldlrap1 | low density lipoprotein receptor adaptor protein 1 | 1,66 | 3,46E-02 | 1,78 | 7,39E-03 | 7,39E-03 |
| Col27a1 | collagen, type XXVII, alpha 1 | 1,60 | 4,29E-02 | 2,09 | 4,85E-03 | 4,85E-03 |
| Hspa4l | heat shock protein 4 like | 1,59 | 4,88E-02 | 1,68 | 1,05E-02 | 1,05E-02 |
| Tmbim1 | transmembrane BAX inhibitor motif containing 1 | 1,58 | 2,12E-02 | 1,80 | 1,87E-03 | 1,87E-03 |
| Slc44a2 | solute carrier family 44, member 2 | 1,58 | 2,98E-02 | 1,50 | 1,35E-02 | 1,35E-02 |
| Parp16 | poly (ADP-ribose) polymerase family, member 16 | 1,57 | 6,99E-03 | 1,62 | 1,51E-02 | 1,51E-02 |
| Col27a1 | collagen, type XXVII, alpha 1 | 1,57 | 4,09E-02 | 2,01 | 3,30E-03 | 3,30E-03 |
| Acp5 | acid phosphatase 5, tartrate resistant | 1,57 | 7,14E-03 | 1,60 | 1,73E-03 | 1,73E-03 |
| A630033H20Rik | RIKEN cDNA A630033H20 gene | 1,56 | 2,13E-02 | 1,53 | 2,32E-02 | 2,32E-02 |
| 1700097N02Rik | RIKEN cDNA 1700097N02 gene | 1,51 | 2,27E-02 | 1,59 | 7,63E-03 | 7,63E-03 |
| Otos | otospiralin | 1,50 | 4,84E-02 | 1,50 | 8,08E-03 | 8,08E-03 |
