# Supplementary material for: NFAT transcription factors are essential and redundant actors for leukemia initiating potential in T-cell acute lymphoblastic leukemia
Source: PLoS One. 2021 Jul 7;16(7):e0254184. doi: 10.1371/journal.pone.0254184 (PMC8263285; doi:10.1371/journal.pone.0254184)

Figure 1B uncropped gels

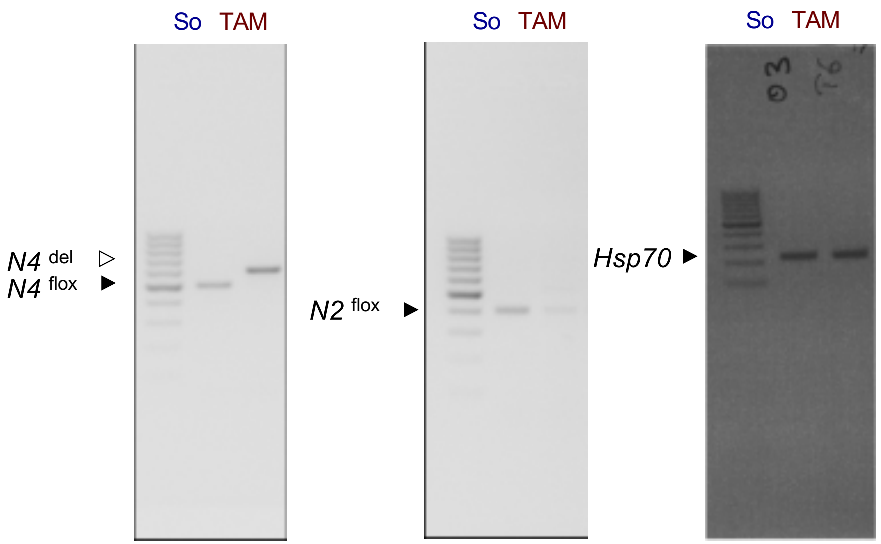

Figure 1C uncropped gels

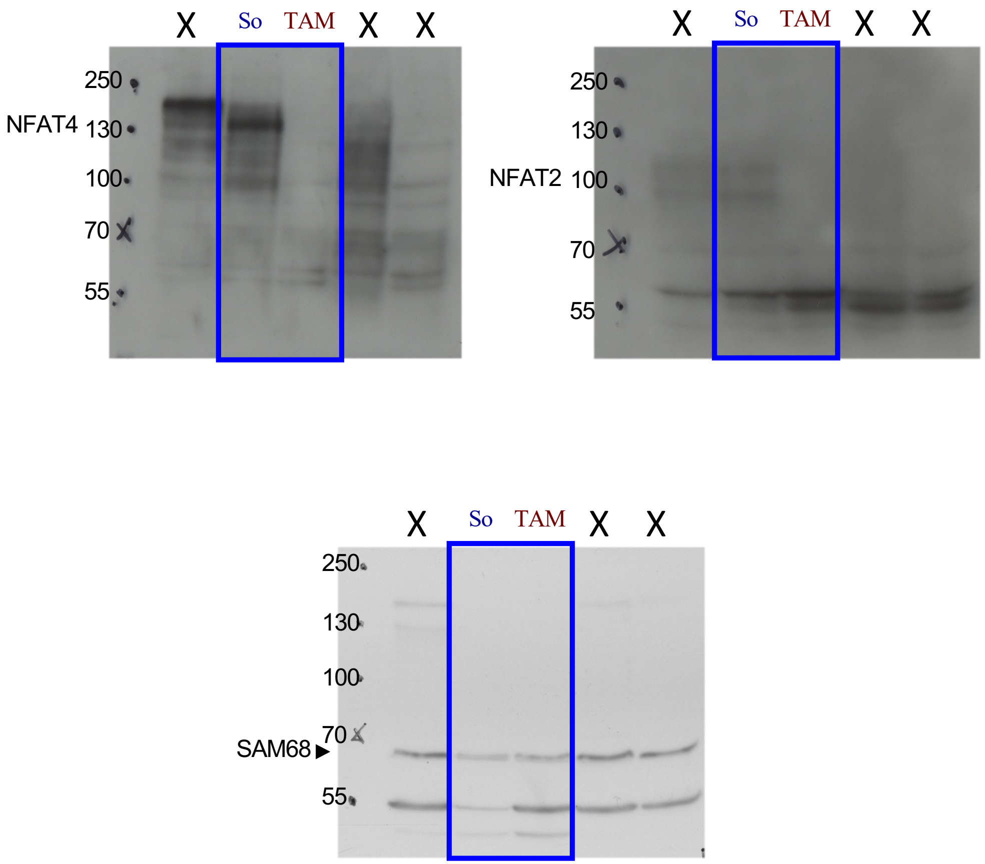

Figure 2A uncropped gels

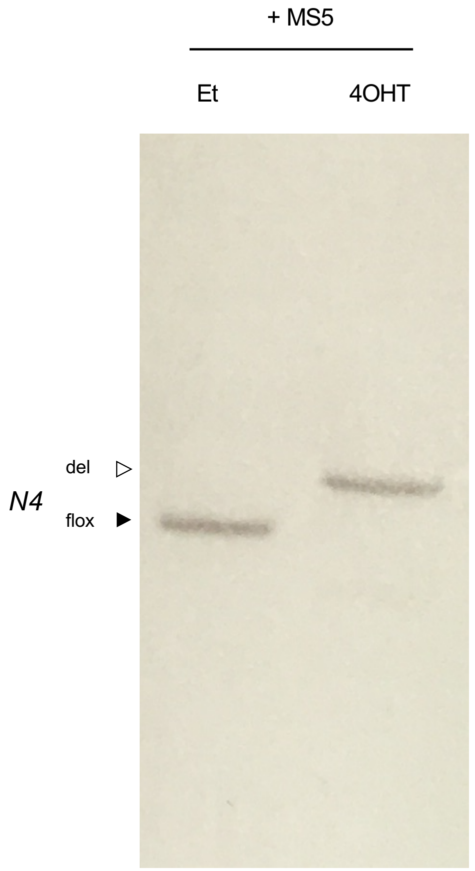

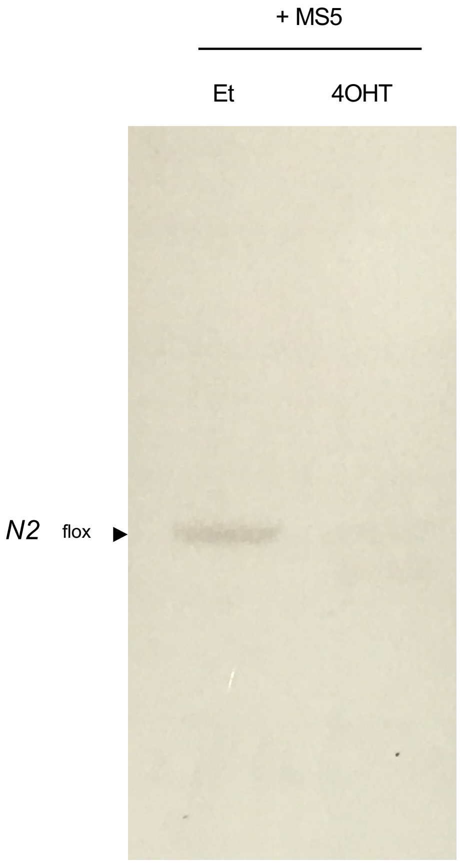

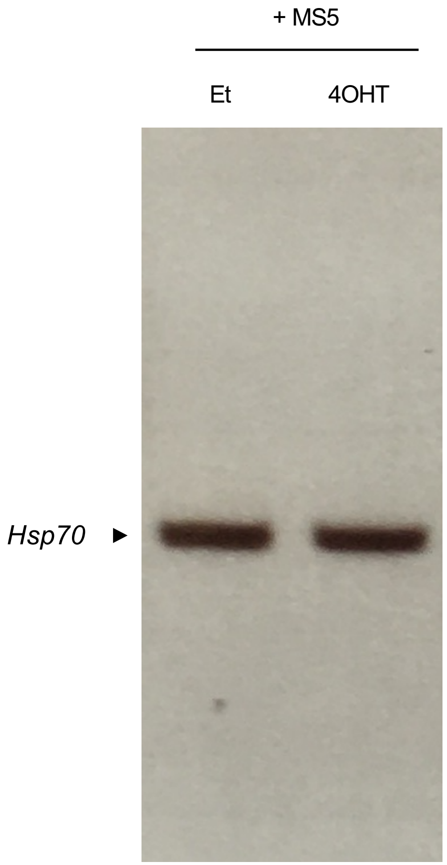

Figure 3B uncropped gels

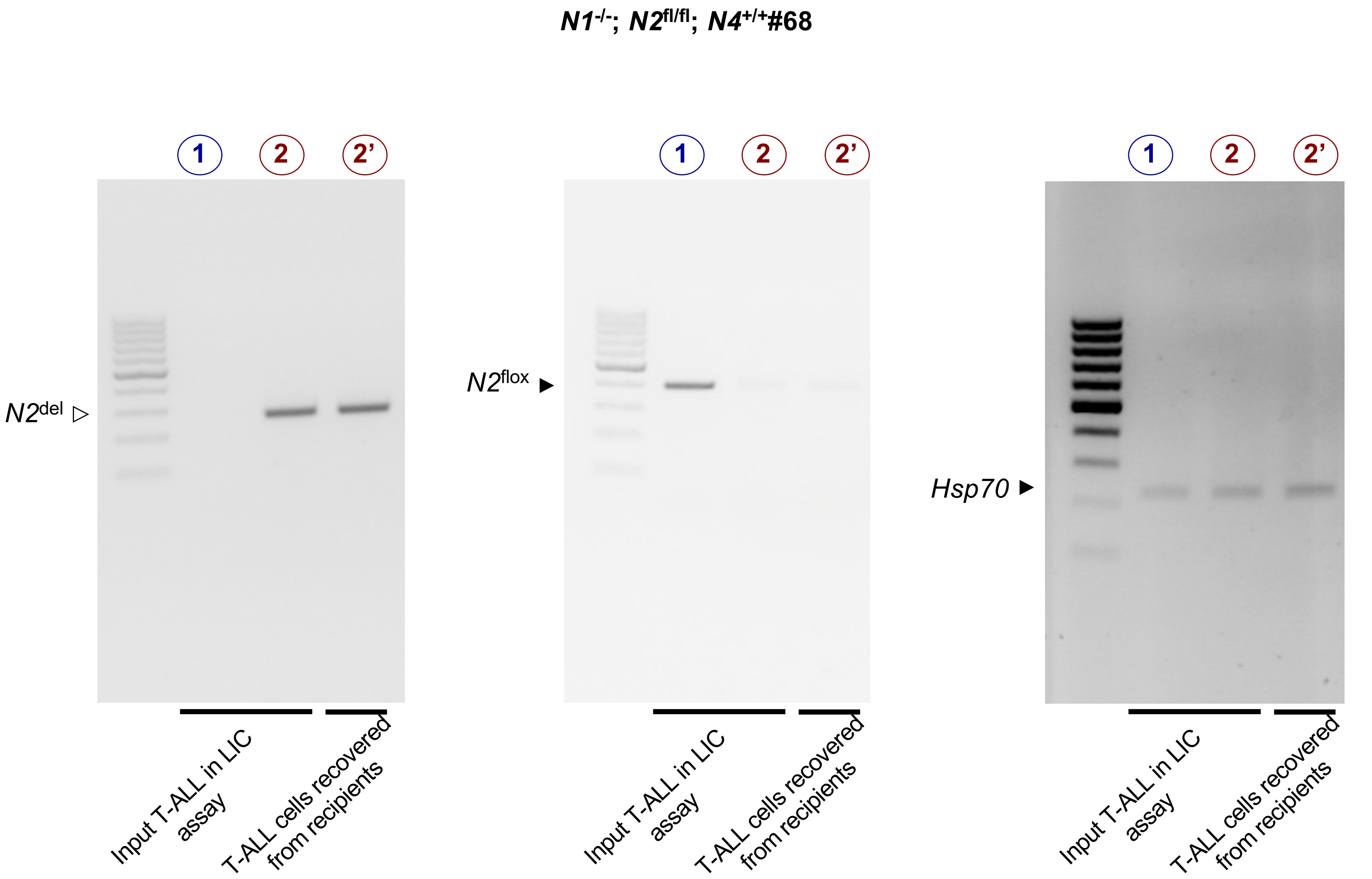

Figure 3B uncropped gels

***N1$^{-/-}$; N2$^{+/+}$; N4$^{fl/fl}$* #14**

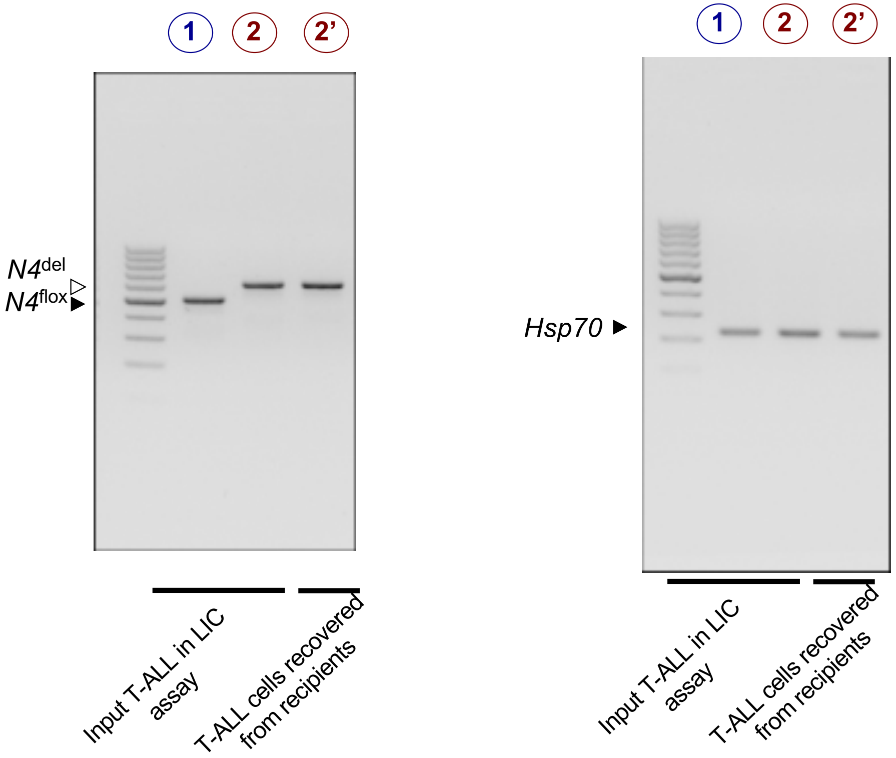

Figure 4B uncropped gels

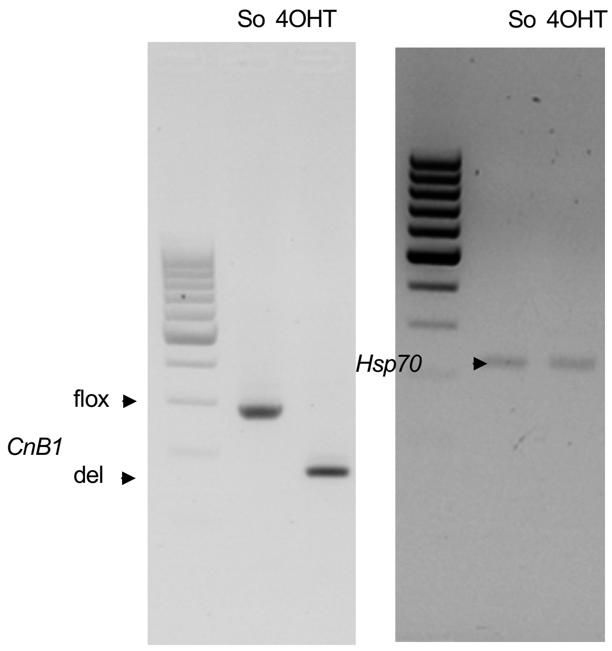

Figure 4D uncropped gels

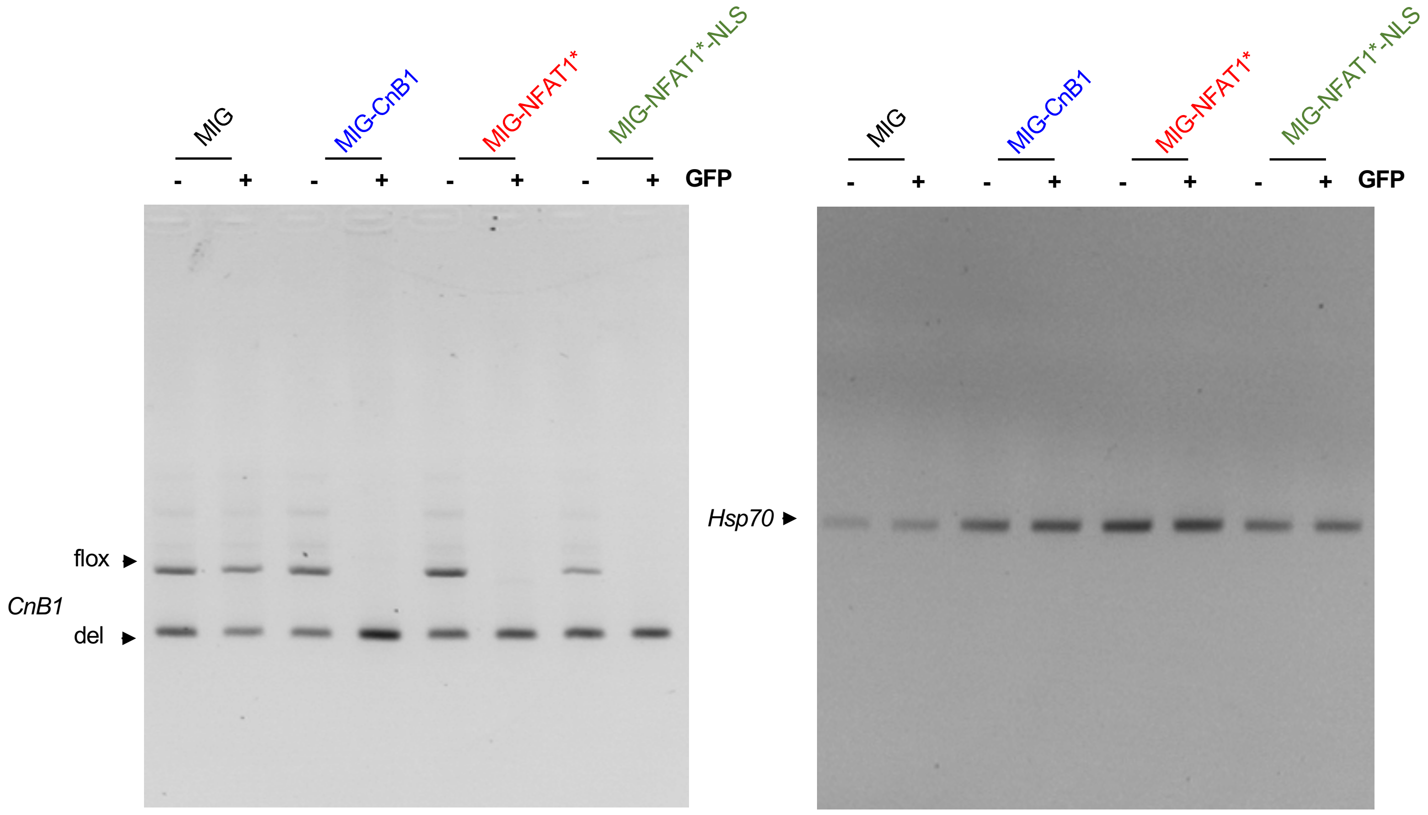

Figure 5D uncropped gels

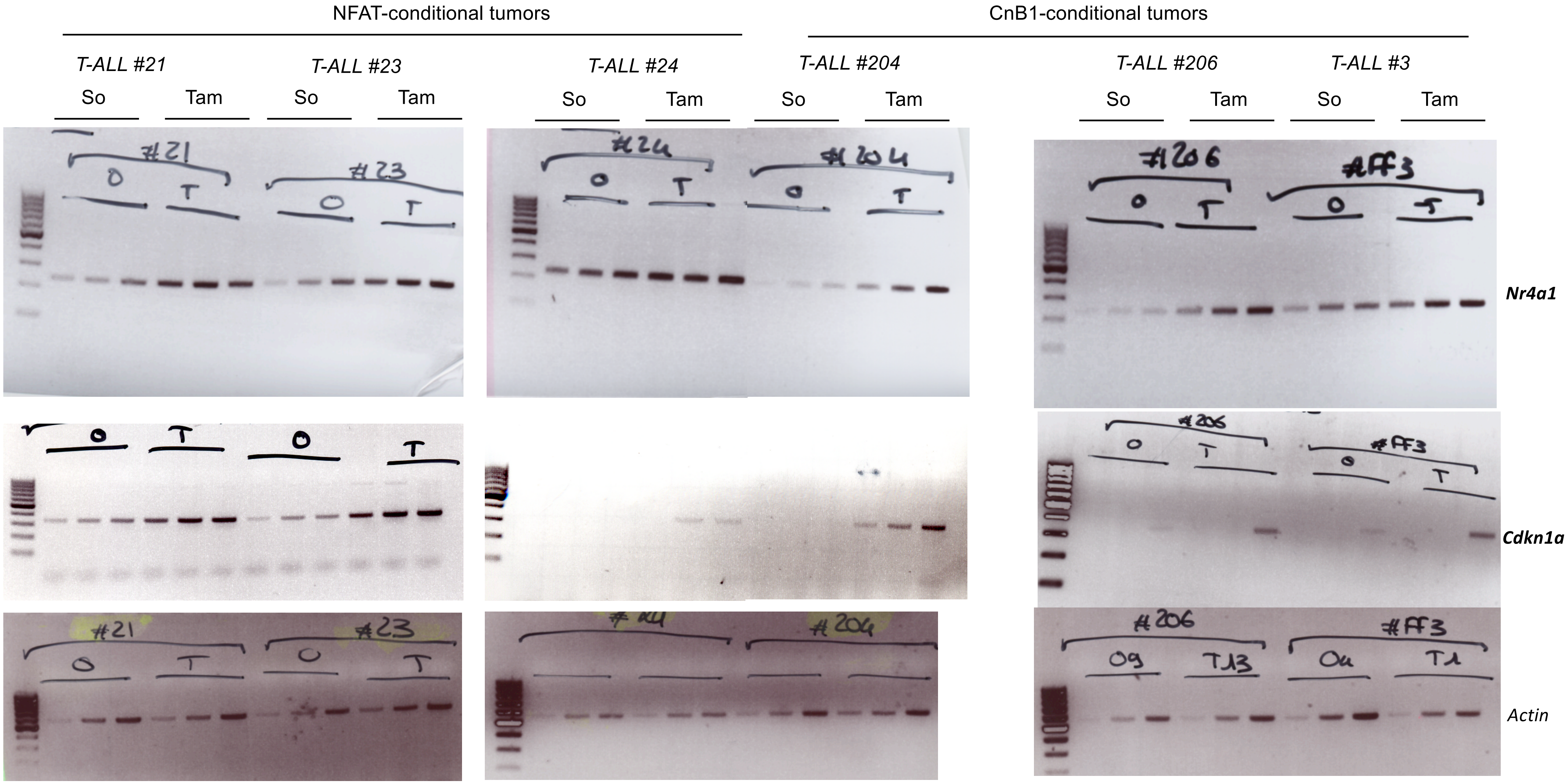

Supplement: S1 Raw images — (PDF) [file pone.0254184.s011.pdf]
